# Computational analysis of the receptor binding specificity of novel influenza A/H7N9 viruses

**DOI:** 10.1186/s12864-018-4461-z

**Published:** 2018-05-09

**Authors:** Xinrui Zhou, Jie Zheng, Fransiskus Xaverius Ivan, Rui Yin, Shoba Ranganathan, Vincent T. K. Chow, Chee-Keong Kwoh

**Affiliations:** 1 School of Computer Science and Engineering, Nanyang Technological University, Singapore, 639798 Singapore; 2 Genome Institute of Singapore, A*STAR, Singapore, 138672 Singapore; 3 Department of Chemistry and Biomolecular Sciences, Macquarie University, Sydney, NSW 2109 Australia; 4 Department of Microbiology and Immunology, Yong Loo Lin School of Medicine, National University of Singapore, Singapore, 117545 Singapore

**Keywords:** Influenza A/H7N9, Host specificity, Receptor binding, Molecular docking, Molecular dynamics simulation

## Abstract

**Background:**

Influenza viruses are undergoing continuous and rapid evolution. The fatal influenza A/H7N9 has drawn attention since the first wave of infections in March 2013, and raised more grave concerns with its increased potential to spread among humans. Experimental studies have revealed several host and virulence markers, indicating differential host binding preferences which can help estimate the potential of causing a pandemic. Here we systematically investigate the sequence pattern and structural characteristics of novel influenza A/H7N9 using computational approaches.

**Results:**

The sequence analysis highlighted mutations in protein functional domains of influenza viruses. Molecular docking and molecular dynamics simulation revealed that the hemagglutinin (HA) of A/Taiwan/1/2017(H7N9) strain enhanced the binding with both avian and human receptor analogs, compared with the previous A/Shanghai/02/2013(H7N9) strain. The Molecular Mechanics - Poisson Boltzmann Surface Area (MM-PBSA) calculation revealed the change of residue-ligand interaction energy and detected the residues with conspicuous binding preference.

**Conclusion:**

The results are novel and specific to the emerging influenza A/Taiwan/1/2017(H7N9) strain compared with A/Shanghai/02/2013(H7N9). Its enhanced ability to bind human receptor analogs, which are abundant in the human upper respiratory tract, may be responsible for the recent outbreak. Residues showing binding preference were detected, which could facilitate monitoring the circulating influenza viruses.

**Electronic supplementary material:**

The online version of this article (10.1186/s12864-018-4461-z) contains supplementary material, which is available to authorized users.

## Background

Influenza A viruses are undergoing continuous and rapid evolution, leading to the concerns about an outbreak or even a pandemic [1]. There have been four major pandemics in recent centuries caused by mutations and reassortment of influenza A viruses, which enabled these pathogens to break the barrier of cross-species transmission and evade the human immune system [2, 3]. As documented by the World Health Organization (WHO), the co-circulating strains of influenza viruses are mainly A/H1N1-pdm09, A/H3N2 and B viruses [4]. In addition, the novel and lethal A/H7N9 has attracted attention with a high case-fatality rate of 40% since the first wave of human infections in late March 2013. At least 489 deaths have been reported to WHO among 1307 laboratory-confirmed human infections as of 16 March 2017 [5]. The unusual increase of infected cases compared to earlier waves since September 2016 has caught the urgent attention of health authorities [6]. The fact that infections of H7N9 in poultry are subclinical makes it challenging to surveil its spreading among poultry and to measure the risk of human infection [7]. Most cases have been identified following their exposure to live birds or live poultry markets. Even with small clusters of human infection cases being detected, the WHO declared low likelihood of human-to-human transmission of avian influenza A/H7N9, with the support of current epidemiological and virological evidence [5].

It is of profound concern that the deadly H7N9 may evolve to enhance its ability to spread among humans, although no sustained evidence has been discovered yet. To date, it is not clear how the mutations of influenza viruses lead to the shift of host specificity, the increase of pathogenicity, and the ability of airborne transmission [8], all of which are taken as requirements for avian influenza to cause pandemics. Great efforts have been made in annotating signatures for cross-species transmission and increased virulence, which can facilitate early detection of potential pandemic strains [9]. However, identifying such genetic markers is under debate, since the adaptation, characterized by positive and repeated selection of mutations, can hardly be distinguished conclusively from other ecological and evolutionary processes that drive the mutations [10]. To address this issue, parallel adaptive animal models are commonly applied to analyzing the process, which can identify repeated and probably gain-of-function mutations [11, 12]. Thus, the parallel adaptive animal models serve as a useful yet expensive tool to provide a basis for understanding host jump. Experiments have shown differential abilities of influenza viruses to bind host-specific sialic acid (SIA) -linked receptors influenced by HA protein [13–15]. However, the mechanisms of adaptive mutation leading to host-specificity shift still need to be elucidated.

With the development of high-throughput sequencing technology and the enriched protein structure databases, it is possible to computationally analyze the sequences and protein structures, and thereby optimize therapies [16]. Su et al. combined molecular docking and molecular dynamics simulation to analyze the conformational changes of drugs bound to neuraminidase (NA) of H7N9 under the mutation *R289K* [17]. Similarly, Pan et al. and Kannan et al. investigated the binding preferences of A/H1N1 HA protein for different host cell receptors, giving insight into the virulence enhanced by the HA mutation D222G [18, 19]. As to the emerging influenza A/H7N9 outbreak, one case declaring no exposure to any live bird, live poultry market or suspicious patient caught our attention (reported on 22 February 2017) [20]. The isolated strain of this patient is A/Taiwan/1/2017(H7N9), denoted as TW17. Here we intend to computationally and systematically investigate this new strain. We conducted both sequence and structure analyses, aiming to explore its potential to gain the ability to spread among humans. Our preliminary sequence analysis shows that the HA protein bears the most mutations among all viral proteins. Therefore, we pointed out the mutations in the HA protein and their corresponding functional domains. Besides, we analyzed the viral binding preference and the residue contributions, giving insight into how the mutations could change the protein structure, especially structures of the functional domains of the protein. First, we generated the HA structure of representative A/H7N9 strains by homology modelling. Then, molecular docking was applied to find the most favorable complexes with avian and human host receptor analogs respectively. Further, molecular dynamics simulation was conducted to reveal the change of residue-ligand interaction induced by the mutations of HA protein. The obtained results are novel and specific to the TW17 strain, providing deeper understanding about the impact of HA mutations and the mechanism of receptor recognition.

## Results and discussion

### Suspicious functional markers and binding regions of the TW17 strain

**Fig. 1:**
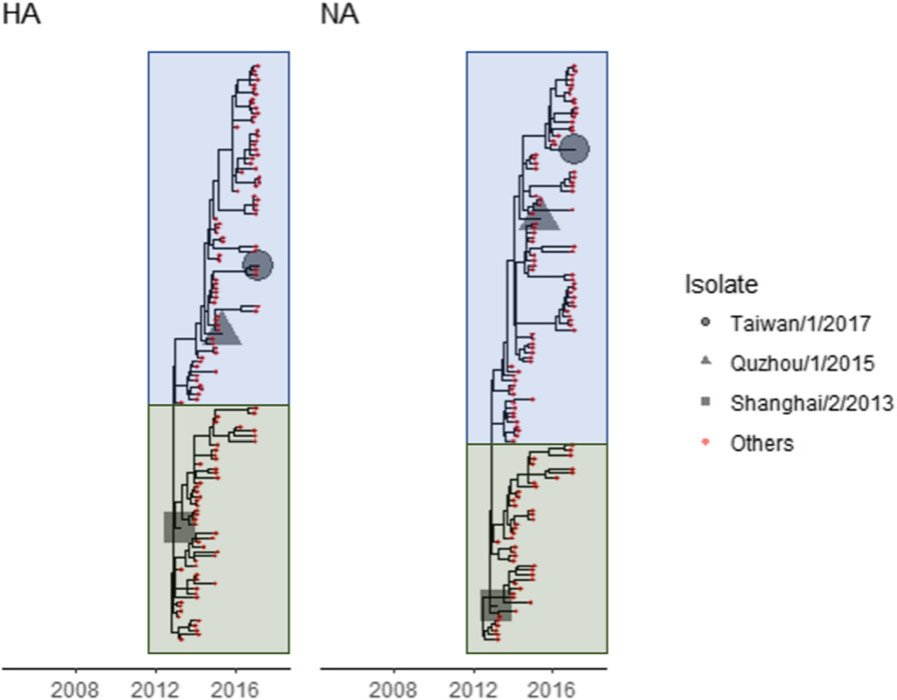
Phylogenetic trees for influenza A/H7N9 HA and NA protein. The blue and green boxes show two major clusters from which HA and NA of recent influenza A/H7N9 emerged

To select representative strains of influenza A/H7N9, we constructed phylogenetic trees for all segments of influenza A/H7N9. We observed that HA and NA of recent influenza A/H7N9 emerged from two major clusters as shown in Fig. 1. A bootstrap-based substitution model showed no conspicuous positive selection in each cluster. Moreover, Fig. 1 indicates that HA and NA of the current circulating strains probably originated from the A/Shanghai/2/2013(H7N9) strain (or SH13 in short). See Additional file 1 for phylogenetic trees of the other segments. Viral protein sequences of the target strain TW17 from the patient who denied any exposure to poultry and live market can be downloaded from the website of Global Initiative on Sharing All Influenza Data (GISAID) [21] by specifying the strain name A/Taiwan/1/2017(H7N9).

We observed four insertions *RKRT* after site 337 and 15 mutations compared to the HA protein of the reference strain SH13. Phenotypically or epidemiologically interesting mutations of the other proteins are categorized by functional impact, summarized in Table 1 with annotations retrieved from FluSurver [22]. Since HA bears the most mutations related with protein functions, we focused on the impact of HA in TW17 strain. Most documented mutations of HA, 14 out of 15, are located at viral oligomerization interfaces, including A130P, S136N, I138T, L235Q and I335V, where the equivalent sites have also been reported as related to antigenic shifts or mild drug resistance [23]. A new potential N-glycosylation site with pattern *NGTR* at sites 136–139 was introduced by the mutations S136N and I138T, and the potential of *NNTY* at site 493 to be N-glycosylated is also likely to increase as predicted by NetNGlyc [24]. It has been reported that glycosylation of HA and NA is associated with host specificity, virulence and human immune response. Thus, glycosylation has been taken as an important way for the influenza viruses to evolve [25].

**Table 1:** Mutations of the influenza TW17 compared to the SH13 strain

Impact	Protein	Mutations
Viral oligomerization interfaces or binding small ligands	HA	I56T, A130P, S136N, I138T, A143V, K182E, L235Q, M245I, A310T, I335V, G338A, E396A, E403K, S499R
	M2	E24D
	NA	M26I, M72I, Y166H, A210V, S242P, R289K, N322S
	PA	G66S
	^a^PB2	M570I, E627K
Host receptor binding	HA	L235Q, E396A
Host specificity shift	PB2	I292V, E627K
Glycosylation	HA	S136 N, I138T
Antibody recognition sites	HA	I56T, A130P, S136 N, I138T, A143V, L235Q, I335V, E396A, S499R
	NA	S242P
Drug binding	NA	S242P, R289K

^a^Best reference hit strain for PB2 is the influenza A/Duck/Guangdong/E1/2012(H10N8)

The results of FluSurver suggested possible significant change of viral binding with small ligands, especially host receptors (Table 1). Furthermore, we applied a carbon probe based approach implemented in SITEHOUND to identify putative ligand binding sites [26]. SITEHOUND calculated an affinity map for the carbon probe and then clustered the points with favorable interaction energies. The cluster with the highest total interaction energy is consistent with known equivalent receptor binding domain (RBD) in H3, including mutations A143V and L235Q which are associated with antibody recognition and host receptor binding. Hence, we used the equivalent RBD of H3 as tentative binding regions to dock host receptor analogs to HA protein, namely 130-loop (139–146), 190-helix (192–204), 220-loop (228–237) and some conserved residues (106, 152, 160–162).

### *Molecular docking predicted the optimal conformations and indicated that the mutant TW17 strain enhanced binding with both* avian receptor analogs *(LSTa) and* human receptor analogs *(LSTc)*

**Fig. 2:**
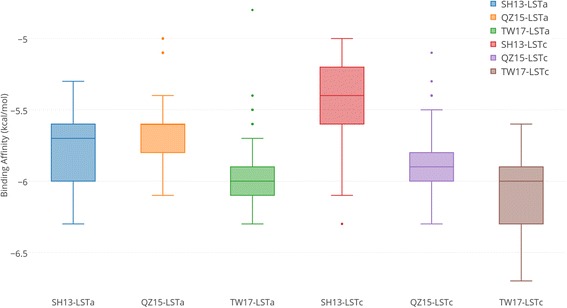
Binding affinity of host receptor analogs with the H7N9 HA proteins. Binding affinity of 500 independent experiments docking LSTa and LSTc to the HA protein of SH13, QZ15 and TW17 strains respectively. SH13-LSTa stands for the docking of LSTa to the HA protein of SH13 strain and so forth

To obtain the optimal complexes of HA protein with host receptors, we conducted molecular docking. LSTa and LSTc are the avian and human receptor analogs respectively. Quick Vina 2 [27] was used to dock the LSTa and LSTc independently with the HA proteins. Besides the target strain TW17, receptor binding properties of two other strains, i.e. the HA of SH13 and A/Quzhou/1/2015(H7N9) (or QZ15 in short), were also investigated for comparison. The SH13 strain was detected as the best reference hit by FluSurver [22], and the HA sequence of QZ15 was the most similar to our target protein which was detected by BLAST as of 31 December 2016 [28]. Each docking experiment was conducted 500 times independently to obtain the best conformation and to analyze the group mean difference. Results of binding affinities for each group of experiments are presented in Fig. 2.

As observed, the HA protein acquired enhanced ability to bind both LSTa and LSTc as the viral strain evolved from SH13 to QZ15 and TW17. The observations were supported by Student’s T-test, shown in Table 2, which was used to assess the significance of group mean difference of docking. Note that LSTc is abundant in human upper respiratory tract, while LSTa, the avian-like cell receptors, is abundant in human lower respiratory tract. The enhanced binding ability to LSTc suggests easier infection to human and a higher potential of airborne transmission, which may partially explain the current epidemic wave.

**Table 2:** T-test for docking experiments (*N* = 500)

Group 1	Group 2	^a^Mean Difference (kcal/mol)	99% Confidence Interval (kcal/mol)	*p*-value (one-tailed)
TW17-LSTa	SH13-LSTa	−0.205	(− 0.237, − 0.173)	< 0.0001
TW17-LSTc	SH13-LSTc	− 0.655	(− 0.696, − 0.614)	< 0.0001
SH13-LSTc	SH13-LSTa	− 0.345	(−0.307, − 0.383)	< 0.0001
TW17-LSTc	TW17-LSTa	−0.105	(− 0.141, − 0.069)	< 0.0001

^a^Mean difference = Mean (Group 1) – Mean (Group 2)

Furthermore, the best and worst conformations were superimposed as shown in Additional file 2. In each HA complex with LSTa, the optimal pose of LSTa has SIA close to the 220-loop, and the worst pose has SIA close to the 130-loop. It is the other way around for the HA complex with LSTc. The optimal pose of LSTc has SIA towards the 130-loop, while the worst pose has SIA towards the 220-loop. The superimposition demonstrates that the docking scores can differentiate reliable poses from non-reliable poses.

However, as tested in [29], docking score functions are good at searching for optimal conformations of ligands, but usually less accurate than atomic scale force fields for describing binding energy. Therefore, the docked complexes with the optimal binding affinity are used to conduct molecular dynamics simulations for further analysis (see Molecular dynamics simulation).

### Molecular dynamics simulation reveals residues that contribute to enhanced binding of HA protein with host cell receptors

**Fig. 3:**
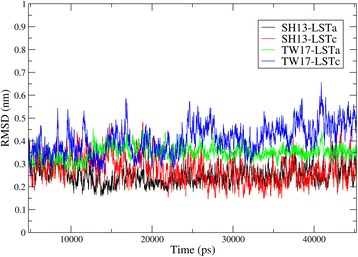
The monitoring of root-mean-square deviation (RMSD) of Cα atoms from the starting coordinates

To observe the dynamic HA-receptor interaction and compare the change of interaction energy induced by substitutions on the protein, we conducted molecular dynamics simulation for the HA proteins to form complexes with receptor analogs. The result of root-mean-square deviation (RMSD) analysis of Cα atoms of HA-LSTa/LSTc complexes from the starting coordinates is shown in Fig. 3. The RMSD indicates that all the trajectories fluctuate within a small range. The TW17-LSTc complex has the largest fluctuation among the four trajectories, but still in a small range (around 0.45 nm).

**Fig. 4:**
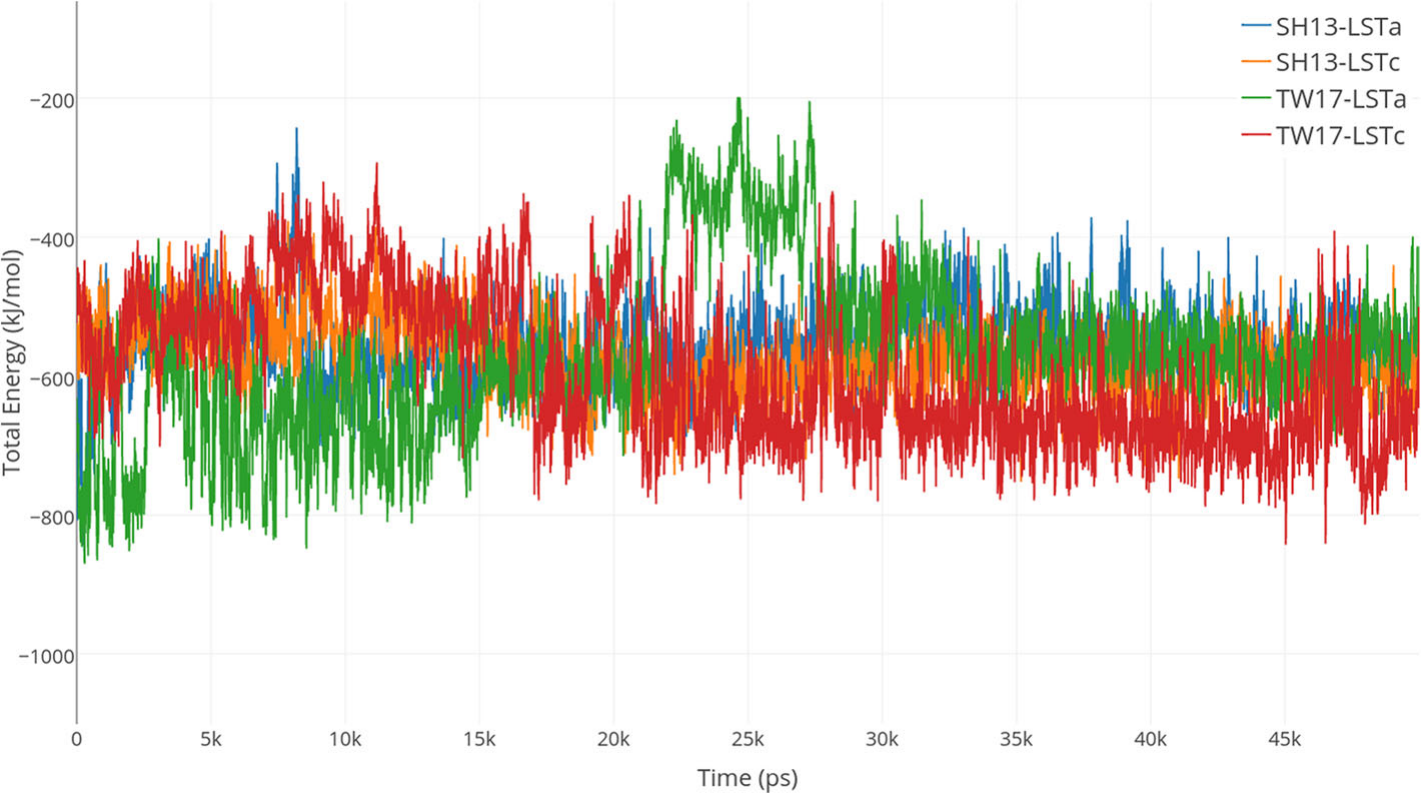
Comparison of HA-SIA vacuum MM total energy. Visualize the fluctuation of total energy and its components for each complex (namely SH13-LSTa, SH13-LSTc, TW17-LSTa and TW17-LSTc) during the whole MD simulation process

The VdW energy, electrostatic energy and total interaction energy of SH13-LSTa, SH13-LSTc, TW17-LSTa and TW17-LSTc are visualized in Additional file 3. The comparison of their total energy is shown in Fig. 4. As observed, the total energy of TW17-LSTa fluctuates obviously during around 25 ns. Each trajectory fluctuated within a small range after 30 ns. Therefore, we chose the last 20 ns frames for further analysis. The average total binding energy values of SH13-LSTa, SH13-LSTc, TW17-LSTa and TW17-LSTc were calculated and listed in Table 3. The mutant TW17 HA obtained the largest binding energy with LSTc (− 664.779 kJ/mol) among the four complexes. In addition, the mutations enhanced binding of HA protein with both LSTa and LSTc. The binding with LSTc was increased by 69.67 kJ/mol, while the binding with LSTa was increased by 13.58 kJ/mol. Both the SH13 and TW17 strains have binding preferences for LSTc. The results may partially explain the outbreak in 2013 and the current epidemic wave in early 2017. To further test this hypothesis, we conducted another 50 ns of molecular dynamics simulation. The results are consistent. The mutant TW17 HA obtains the largest binding energy, and enhances the binding with two types of receptors, especially LSTc. Both SH13 and TW17 strains have binding preferences to LSTc. Additional file 4 shows the simulation results of each system and the average total binding energy.

**Table 3:** Average total binding energy (kJ/mol) of the HA-LSTa/LSTc complexes

	LSTa	LSTc	^a^ΔE_1_
SH13	− 541.559	− 595.111	+ 53.553
TW17	− 555.135	- 664.779	+ 109.644
^b^ΔE_2_	+ 13.576	+ 69.667	

^a^Binding preference of HA protein: ΔE_1_ = ΔE_HA, LSTa_ – ΔE_HA, LSTc_

^b^Difference of HAs binding to receptors: ΔE_2_ = ΔE_SH13, receptor_ – ΔE_TW17, receptor_

**Fig. 5:**
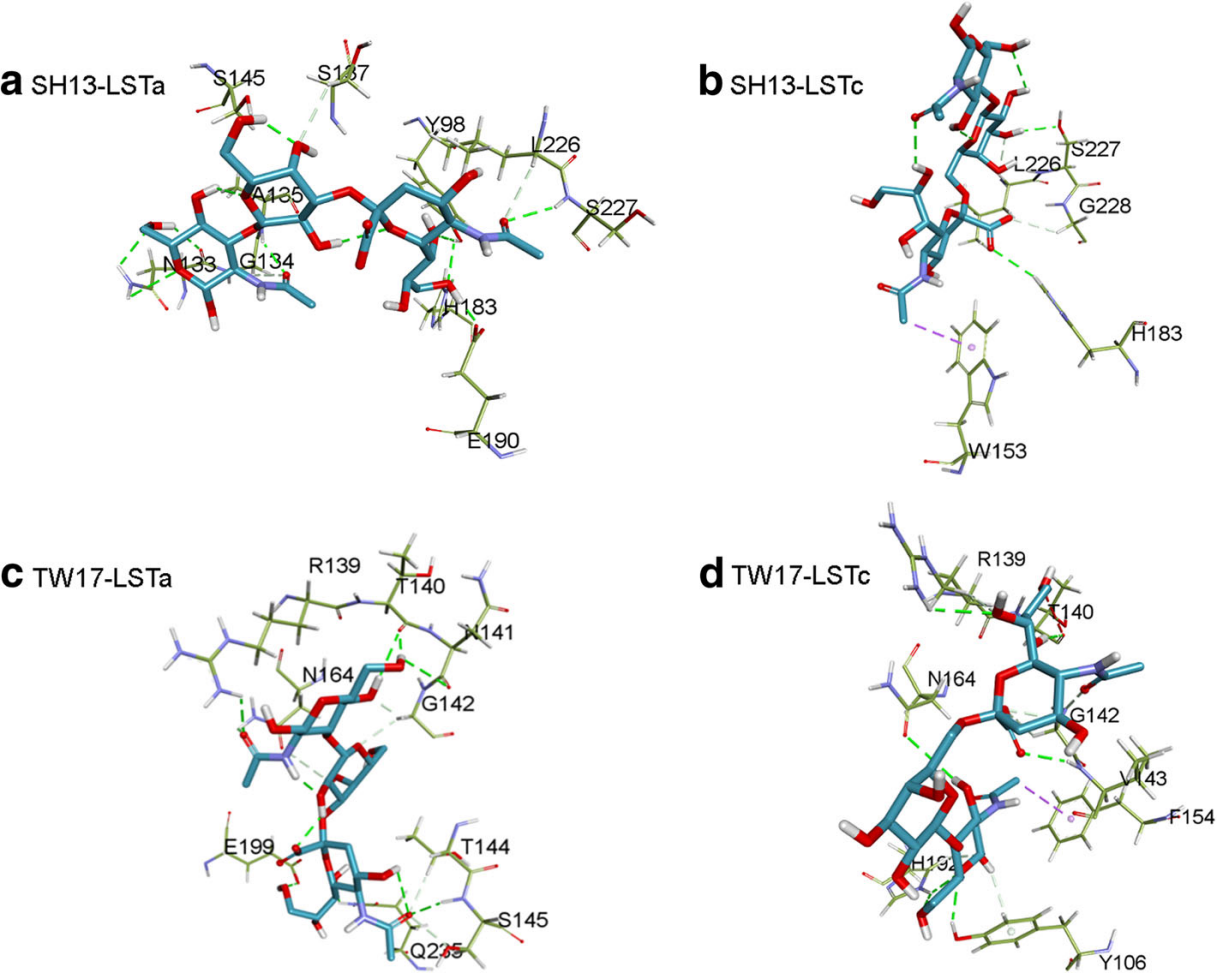
The receptor-ligand interactions in the optimally docked complexes. **a** The optimally docked SH13-LSTa complex. **b** The optimally docked SH13-LSTc complex. **c** The optimally docked TW17-LSTa complex. **d** The optimally docked TW17-LSTc complex

To analyze the residue contribution to the enhanced binding, we visualize the receptor-ligand interactions in the optimally docked complexes in Fig. 5. For the SH13 strain, H192, L235 and S236 interacted with both LSTa and LSTc, the numbers have been converted to equivalent sites in the TW17 HA protein. For the TW17 strain, residues R139, T140, G142 and N164 interact with both LSTa and LSTc. Residue A143 in the HA of SH13 interacts with LSTa, but in the HA of TW17, V143 interacts with LSTc.

**Fig. 6:**
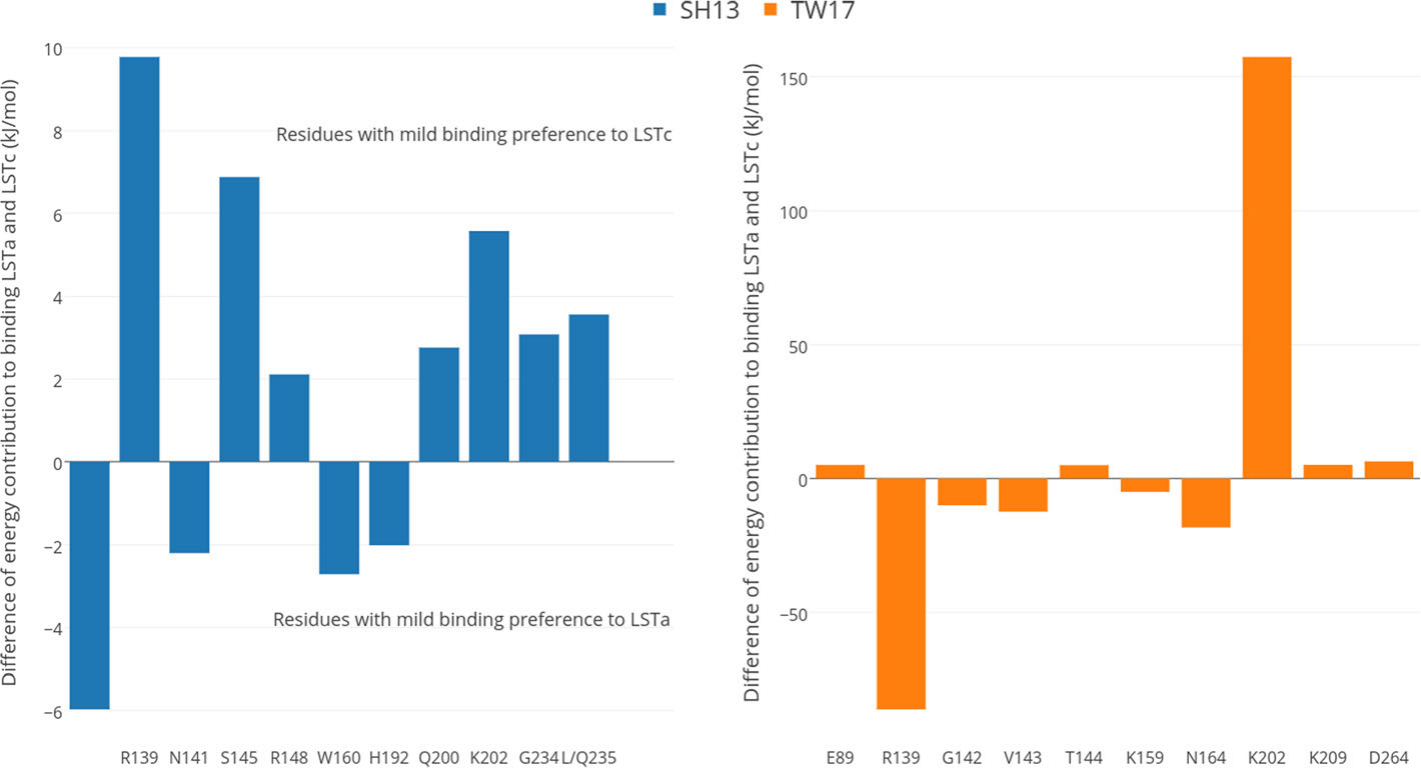
The top 10 residues in HA that show binding preference for LSTa or LSTc

**Fig. 7:**
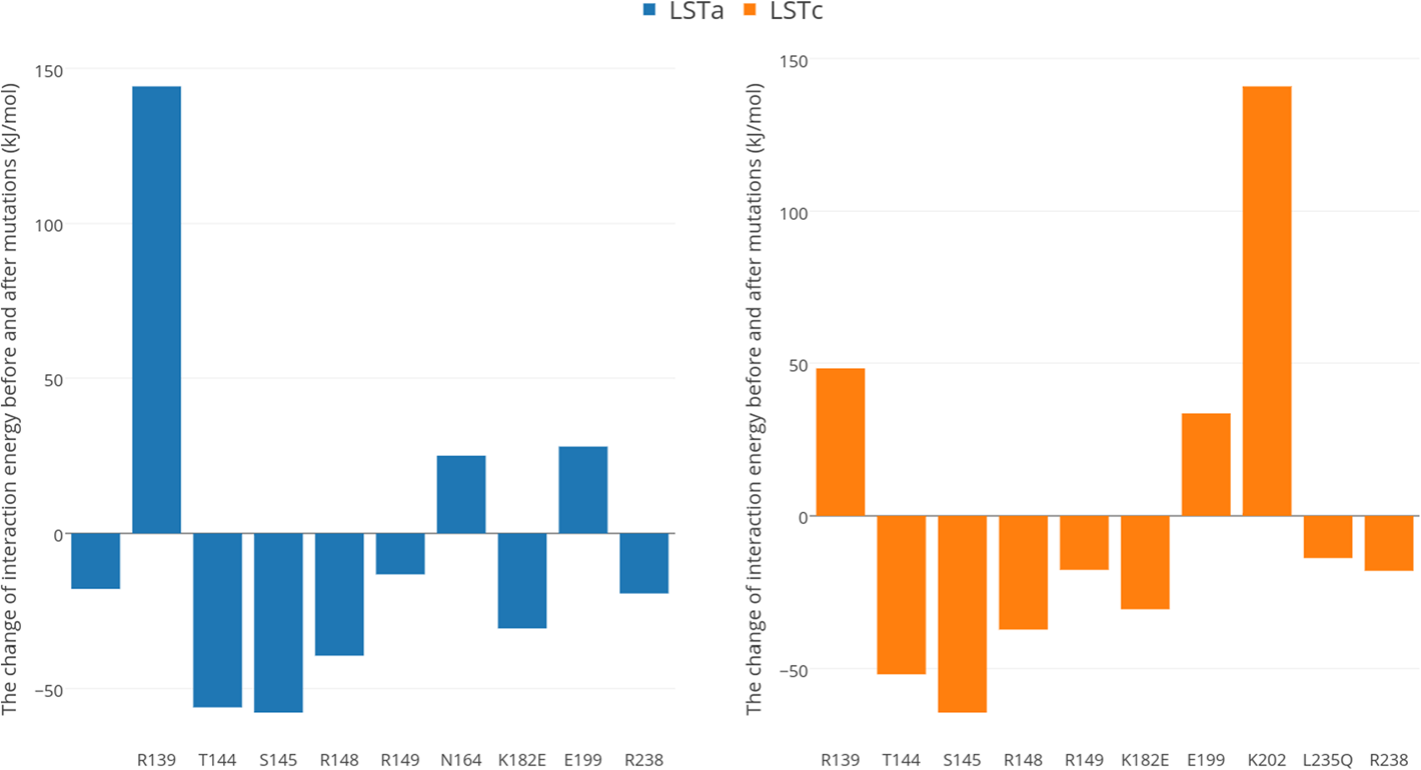
The top 10 residues in HA that affect the HA binding with LSTa or LSTc

Furthermore, we decomposed the total interaction energy to observe the contribution of each residue. The convergence of energy contributions of the residues involved in interaction is shown in Additional file 5. To be consistent with the analysis of total energy, we also used the last 20 ns frames. The average contribution of each residue to the total binding energy was calculated. We focused on the residues that are involved in receptor-ligand interactions in the optimally docked complexes. Additional file 6 shows their average energy contribution in each complex. R139 enhances the binding of HA of TW17 strain with both LSTa and LSTc and it has binding preference for LSTa. Similarly, we quantified the binding preference of all residues by calculating the difference between energy contribution to binding LSTa and LSTc (ΔΔG = ΔG_HA-LSTa_ – ΔG_HA-LSTc_). For the HA of SH13 strain, the differences of energy contribution are all less than 10 kJ/mol. For instance, R139 had mild preference for LSTc. In contrast to the HA of SH13 strain, the mutant TW17 HA protein contains more residues showing apparent binding preference, including R139, V143, N164 (preferring LSTa) and K202 (preferring LSTc). The top 10 residues showing binding preference are visualized in Fig. 6. To investigate the impact of mutations on energy contribution, we also calculated the change of interaction energy before and after the mutations (ΔΔG = ΔG_SH13-receptor_ – ΔG_TW17-receptor_). The top 10 residues that affect the binding with receptors are presented in Fig. 7. R139 and K202 largely enhanced (with ΔΔG > 100 kJ/mol) the binding the LSTa and LSTc respectively. Note that R139 is located at a new potential N-glycosylation sites introduced by the mutations S136 N and I138T, which may explain the increased binding with receptors. E199 and K202, which located at the 190-helix, have enhanced the binding with LSTc, but no mutation is observed in this region. The mutations nearby the 190-helix might be responsible for the change of residue contributions.

## Conclusions

In this study, we analyzed the mutations of the current circulating influenza A/H7N9 virus strain, isolated from a patient claiming no exposure to any live bird, live poultry market or suspicious patient. We highlighted mutations bearing critical protein functions, namely host receptor binding, drug binding, antibody recognition and glycosylation. Furthermore, we focused on the HA binding with different host cell receptors by performing molecular docking and molecular dynamics simulation. The obtained results indicate that the mutant HA enhances its binding with both avian and human receptor analogs, especially human receptor analogs. Also, the MM-PBSA calculations of residue-ligand interaction revealed several critical residues showing binding preference, including residues R139, V143, N164 and K202. Similarly, major residues contributing to the change of interaction energy between HA and receptors were highlighted. We detected the mutations S136 N, I138T and mutations near the 190-helix as the most important substitutions in the HA protein. Although S136N, I138T do not cause direct change to the interaction energy, they introduce a new glycosylation pattern. Besides, their neighboring residue R139 enhances binding to both avian and human receptors.

The obtained results are novel and specific to the influenza A/Taiwan/1/2017(H7N9) strain, shedding light on the impacts of HA mutations and the mechanisms of receptor recognition. In addition, our pipeline of analysis should be applicable to analyzing the impacts of other mutations on the binding of proteins with small ligands.

## Methods

### Data

All protein sequences of influenza A/H7N9 were retrieved from Global Initiative on Sharing All Influenza Data (GISAID) EpiFlu Database [21] as of 8 April 2017. The virus strain in the patient claiming no exposure to any live bird or any live poultry market is influenza A/Taiwan/1/2017(H7N9) (Isolate ID: EPI_ISL_248778), denoted as TW17. HA of the strain from the first wave A/Shanghai/02/2013(H7N9) and the most similar HA sequence from A/Quzhou/1/2015(H7N9) were selected as references, denoted as SH13 and QZ15 respectively. The three representative strains were used to docking with host receptor analogs to show the changes of HA binding preference. The accession IDs are YP_009118475 and AKI82233 for HA proteins of SH13 and QZ15 respectively.

To conduct protein-ligand docking and molecular dynamics simulation, we need the structures of HA proteins and receptor analogs. Examining the Protein Data Bank (PDB), we found crystal structures of SH13 HA with LSTa (PDB ID: 4N5K) and LSTc (PDB ID:4N60) [30]. However, extra four residues were observed in the HA protein of the new TW17 strain, by comparing it with the sequences of available HA structures in PDB. Hence, we obtained the HA structures of the other representatives from homology modelling described in the next section. Avian and human receptor analogs were obtained from PDB:5E2Z [31] and 2YP3 [32] respectively.

### Homology modelling

We used SWISS-MODEL for the homology modelling, constructing an atomic-resolution model of the target HA protein from its amino acid sequence [33, 34]. First, the primary sequence of the HA protein from the TW17 strain was searched with BLAST [35] and HHBlits [36] against the SWISS-MODEL template library [37], finding 1218 templates. Three templates with the highest quality were selected, two of which were found by HHBlits (PDBID: 3WHE.1.A and 4LN6.1.A) and one found by BLAST (PDBID: 4WSW.1.F). Models were built based on the target-template alignment using ProMod3 and measured with Global Model Quality Estimation (GMQE), a quality estimation combining properties from the target-template alignment while considering the QMEAN score of the obtained model [38, 39]. The model constructed from template 3WHE.1.A had the highest GMQE value of 0.72. Thus, it was selected for further analysis.

### Phylogenetic tree construction

For phylogenetic tree analysis of PB2, PB1, PA, HA, NP, NA, M1 and NS1 genes of influenza H7N9 strains, 125 available isolates were collected (two genomes, whose isolate IDs are EPI ISL 148744 and EPI ISL 142188, were removed due to quality considerations). For each gene, the coding sequences (CDSs) were obtained from each isolate using influenza virus sequence annotation tool provided by NCBI [40], and then the CDSs were aligned to codon positions using MUSCLE [41]. Then, the Bayesian Markov Chain Monte Carlo (MCMC) implemented in Beast v2.4.4 [42] was used to infer a time-tree from the alignment. The Beast software was run under the HKY substitution model, a Bayesian skyline coalescence model, and a strict molecular clock model to produce 2000 tree samples logged every 25,000 generations. Beast’s outputs were assessed with TRACER v1.6 [43] and summarized as a maximum clade credibility (MCC) tree using TreeAnnotator v2.4.4 with 10% burn-in. The time-tree for each gene was visualized using ggtree package [44].

### Protein-ligand docking

Molecular docking is commonly used to estimate the pose of conformation and roughly compare the binding affinity of a small number of complexes. With the structures of HA and host cell receptor analogs obtained, we applied protein-ligand docking using QuickVina 2, which optimized the local search of docked conformation candidates by a novel first-order-consistency-check heuristic [27]. All available rotatable bonds of receptor analogs, LSTa and LSTc, were activated to ensure flexibility. They were docked respectively to the receptor binding domain of the H7N9 HA protein (sites 106, 139–146, 152, 160–162, 192–204, 228–237). The top conformation with the optimal binding affinity among 500 independent docking experiments was selected to analyze the interactions between the host receptor analogs and the HA proteins.

### Molecular dynamics simulation

The complexes of SH13 and TW17 HA proteins with LSTa and LSTc were used in molecular dynamics simulation. The complexes SH13-LSTa and SH13-LSTc were obtained from PDB (PDB ID: 4N5K and 4N60), while complexes TW17-LSTa and TW17-LSTc were from the optimal docking results.

GROMACS 5.1.2 [45] was used in the whole simulation process, energy minimization and equilibration of the system involved. The AMBER99SB-ILDN force field was used to describe the system [46]. All complexes were solvated by using the explicit TIP3P water model in a cubic box and counter ions were added to neutralize the system [47]. Steepest-descent energy minimization was applied to each complex. Position restrains for both NVT and NPT equilibration were conducted for 100 ps with modified Berendsen thermostat and Parrinello-Rahman pressure coupling [48, 49]. Temperature, pressure, density and total energy were all well equilibrated before running the production MD simulation for 50 ns.

### Binding free energy and residue-ligand interaction energy calculation

Snapshots of MD simulations were recorded to analyze the binding free energy. We applied Molecular Mechanics - Poisson Boltzmann Surface Area (MM-PBSA) to estimate the binding free energy ΔG_bind_ under the assumption of no configurational rearrangement upon which the free energy changes. It is computed as:

1 $$ {\Delta \mathrm{G}}_{\mathrm{bind}}={\Delta \mathrm{G}}_{\mathrm{bind},\mathrm{vac}}+{\Delta \mathrm{G}}_{\mathrm{bind},\mathrm{solv}} $$

The binding free energy ΔG_bind_ comprises the binding free energy in the vacuum phase ΔG_bind, vac_ and the solvation free energy ΔG_bind, solv_, which is the difference of solvation free energy values of complex, receptor and ligand:

2 $$ {\Delta \mathrm{G}}_{\mathrm{bind},\mathrm{solv}}={\Delta \mathrm{G}}_{\mathrm{solv},\mathrm{complex}}\hbox{--} \left({\Delta \mathrm{G}}_{\mathrm{solv},\mathrm{receptor}}+{\Delta \mathrm{G}}_{\mathrm{solv},\mathrm{ligand}}\right) $$

The solvation free energy for each component is composed of polar and non-polar energy derived from the PB equation and the SA method, computed as:

3 $$ {\Delta \mathrm{G}}_{\mathrm{solv}}={\Delta \mathrm{G}}_{\mathrm{solv},\mathrm{polar}}+{\Delta \mathrm{G}}_{\mathrm{solv},\mathrm{nonpolar}} $$

The binding free energy and decomposed interaction energy between residues of HA protein and receptor analogs were calculated using the g_mmpbsa package [50].

## Additional files

Additional file 1: Phylogenetic trees of influenza A/H7N9. Time-scale phylogenetic trees of PB2, PB1, PA, HA, NP, NA, M1 and NS1 genes of influenza H7N9 strains. (PDF 117 kb)

Additional file 2: Superimpose the best and worst docked HA-ligand complexes. Visualize the structure of docked SH13-LSTa, SH13-LSTc, TW17-LSTa and TW17-LSTc complexes with the highest and the lowest binding affinities. (PDF 466 kb)

Additional file 3: Energy decomposition of HA-ligand complexes. VdW energy, electrostatic energy and total interaction energy of SH13-LSTa, SH13-LSTc, TW17-LSTa and TW17-LSTc. (PDF 702 kb)

Additional file 4: Two rounds of 50 ns molecular dynamics simulation for SH13-LSTa, SH13-LSTc, TW17-LSTa, TW17-LSTc. Average total binding energy (kJ/mol) of the HA-LSTa/LSTc complexes. (PDF 424 kb)

Additional file 5: Residues contribution during the whole simulation process. The convergence analysis of energy contribution for residues involved in HA-ligand interaction. (PDF 153 kb)

Additional file 6: Average energy contribution of residues that involved in receptor-ligand interactions in the optimally docked complexes. (PDF 24 kb)

## Abbreviations

A/H1N1-pdm09: The 2009 pandemic strains of Influenza A/H1N1
GMQE: Global model quality estimation
HA: Hemagglutinin
LSTa: Avian receptor analogs with α(2,3) linked SIA
LSTc: Human receptor analogs with α(2,6) linked SIA
MM-PBSA: Molecular mechanics - poisson boltzmann surface area
NA: Neuraminidase
QZ15: Influenza A/Quzhou/1/2015(H7N9)
RMSD: Root-mean square deviation
SH13: Influenza A/Shanghai/02/2013(H7N9)
SIA: Sialic acid
TW17: Influenza A/Taiwan/1/2017(H7N9)
WHO: World Health Organization

## References

[1] Tscherne DM, García-Sastre A (2011). Virulence determinants of pandemic influenza viruses. J Clin Invest.

[2] Smith GJD (2009). Dating the emergence of pandemic influenza viruses. Proc Natl Acad Sci.

[3] Viboud C, Simonsen L (2012). Global mortality of 2009 pandemic influenza a H1N1. Lancet Infect Dis.

[4] World Health Organization. Recommended composition of influenza virus vaccines for use in the 2016–2017 northern hemisphere influenza season. 2016; Available from: http://www.who.int/influenza/vaccines/virus/recommendations/en/ (accessed 17 Mar 2017).

[5] World Health Organization. Monthly risk assessment summary*.* 2017; Available from: http://www.who.int/influenza/human_animal_interface/HAI_Risk_Assessment/en/ (accessed 23 Apr 2017).

[6] Shen Y, Lu H. Global concern regarding the fifth case of human infection with avian influenza a (H7N9) virus in China. Bioscience Trends. 2017;11.1:120–21 10.5582/bst.2017.01040 28250340

[7] Xiang N. Assessing change in avian influenza a (H7N9) virus infections during the fourth epidemic—China, September 2015--august 2016. MMWR Morb Mortal Wkly Rep. 2016;65 10.15585/mmwr.mm6549a2 27977644

[8] Taubenberger JK, Kash JC (2010). Influenza virus evolution, host adaptation, and pandemic formation. Cell Host Microbe.

[9] Tumpey TM (2007). A two-amino acid change in the hemagglutinin of the 1918 influenza virus abolishes transmission. Science.

[10] Pepin KM (2010). Identifying genetic markers of adaptation for surveillance of viral host jumps. Nat Rev Microbiol.

[11] Ping J (2011). Genomic and protein structural maps of adaptive evolution of human influenza a virus to increased virulence in the mouse. PLoS One.

[12] Narasaraju T (2009). Adaptation of human influenza H3N2 virus in a mouse pneumonitis model: insights into viral virulence, tissue tropism and host pathogenesis. Microbes Infect.

[13] Ibricevic A (2006). Influenza virus receptor specificity and cell tropism in mouse and human airway epithelial cells. J Virol.

[14] Xiong X, McCauley JW, Steinhauer DA (2014). Receptor binding properties of the influenza virus hemagglutinin as a determinant of host range. Current Topics Microbiol Immunol.

[15] Edinger TO, Pohl MO, Stertz S (2014). Entry of influenza a virus: host factors and antiviral targets. J Gen Virol.

[16] Koday MT (2016). A computationally designed hemagglutinin stem-binding protein provides in vivo protection from influenza independent of a host immune response. PLoS Pathog.

[17] Su CT-T (2013). Structural analysis of the novel influenza a (H7N9) viral neuraminidase interactions with current approved neuraminidase inhibitors Oseltamivir, Zanamivir, and Peramivir in the presence of mutation R289K. BMC Bioinformatics.

[18] Pan D (2012). Molecular mechanism of the enhanced virulence of 2009 pandemic influenza a (H1N1) virus from D222G mutation in the hemagglutinin: a molecular modeling study. J Mol Modeling.

[19] Kannan S, Kolandaivel P (2016). Computational studies of pandemic 1918 and 2009 H1N1 hemagglutinins bound to avian and human receptor analogs. J Biomol Struct Dyn.

[20] World Health Organization. Human infection with avian influenza a(H7N9) virus - China (22-Feb-2017)*.* 2017; Available from: http://www.who.int/csr/don/22-february-2017-ah7n9-china/en/ (accessed 27 Feb 2017).

[21] Büch J (2011). Gisaid-a global initiative on sharing all influenza data. Influenza Other Respir Viruses.

[22] A*STAR Bioinformatics Institute. *Influenza surveillance- prepared for the next wave*. Available from: http://flusurver.bii.a-star.edu.sg/ (accessed 17 Mar 2017).

[23] Philpott M (1990). Hemagglutinin mutations related to attenuation and altered cell tropism of a virulent avian influenza a virus. J Virol.

[24] Gupta R, Brunak S. Prediction of glycosylation across the human proteome and the correlation to protein function. Pac Symp Biocomput. 2001;7:310–22. 11928486

[25] Wu C-Y (2017). Influenza A surface glycosylation and vaccine design. Proc Natl Acad Sci.

[26] Hernandez M, Ghersi D, Sanchez R (2009). SITEHOUND-web: a server for ligand binding site identification in protein structures. Nucleic Acids Res.

[27] Alhossary A (2015). Fast, accurate, and reliable molecular docking with QuickVina 2. Bioinformatics.

[28] Johnson M (2008). NCBI BLAST: a better web interface. Nucleic Acids Res.

[29] Ramírez D, Caballero J (2016). Is it reliable to use common molecular docking methods for comparing the binding affinities of enantiomer pairs for their protein target?. Intl J Mol Sci.

[30] Xu R (2013). Preferential recognition of avian-like receptors in human influenza a H7N9 viruses. Science.

[31] Rose AS (2016). Web-based molecular graphics for large complexes. Proceedings of the 21st international conference on Web3D technology.

[32] Lin YP (2012). Evolution of the receptor binding properties of the influenza a (H3N2) hemagglutinin. Proc Natl Acad Sci.

[33] Biasini M, et al. SWISS-MODEL: modelling protein tertiary and quaternary structure using evolutionary information. Nucleic Acids Res. 2014;42(W1):W252–W258. 10.1093/nar/gku340 PMC4086089 24782522

[34] Arnold K (2006). The SWISS-MODEL workspace: a web-based environment for protein structure homology modelling. Bioinformatics.

[35] Altschul SF (1997). Gapped BLAST and PSI-BLAST: a new generation of protein database search programs. Nucleic Acids Res.

[36] Remmert M (2012). HHblits: lightning-fast iterative protein sequence searching by HMM-HMM alignment. Nat Methods.

[37] Kiefer F (2009). The SWISS-MODEL repository and associated resources. Nucleic Acids Res.

[38] Benkert P, Biasini M, Schwede T (2010). Toward the estimation of the absolute quality of individual protein structure models. Bioinformatics.

[39] Šali A, Blundell TL (1993). Comparative protein modelling by satisfaction of spatial restraints. J Mol Biol.

[40] Bao Y (2007). FLAN: a web server for influenza virus genome annotation. Nucleic Acids Res.

[41] Edgar RC (2004). MUSCLE: multiple sequence alignment with high accuracy and high throughput. Nucleic Acids Res.

[42] Bouckaert R (2014). BEAST 2: a software platform for Bayesian evolutionary analysis. PLoS Comput Biol.

[43] Rambaut, A., et al. *Tracer v1.6*. Tracer (online 2015, may 29) 2014; Available from: http://beast.bio.ed.ac.uk (accessed 13 Apr 2017).

[44] Yu G (2017). Ggtree: an r package for visualization and annotation of phylogenetic trees with their covariates and other associated data. Methods Ecol Evol.

[45] Abraham MJ (2015). GROMACS: high performance molecular simulations through multi-level parallelism from laptops to supercomputers. SoftwareX.

[46] Lindorff-Larsen K (2010). Improved side-chain torsion potentials for the amber ff99SB protein force field. Proteins: Structure, Function, Bioinformatics.

[47] Jorgensen WL (1983). Comparison of simple potential functions for simulating liquid water. J Chem Phys.

[48] Berendsen HJC (1984). Molecular dynamics with coupling to an external bath. J Chem Phys.

[49] Parrinello M, Rahman A (1981). Polymorphic transitions in single crystals: a new molecular dynamics method. J Appl Phys.

[50] Kumari R, Kumar R, Lynn A (2014). g_mmpbsa: a GROMACS tool for high-throughput MM-PBSA calculations. J Chem Inf Model.

