# Supplementary material for: Computational analysis of the receptor binding specificity of novel influenza A/H7N9 viruses
Source: BMC Genomics. 2018 May 9;19(Suppl 2):88. doi: 10.1186/s12864-018-4461-z (PMC5954268; doi:10.1186/s12864-018-4461-z)
Supplement: Supplementary file 1 — Phylogenetic trees of influenza A/H7N9. Time-scale phylogenetic trees of PB2, PB1, PA, HA, NP, NA, M1 and NS1 genes of influenza H7N9 strains. (PDF 117 kb) [file 12864_2018_4461_MOESM1_ESM.pdf]

Additional file 1: Phylogenetic trees of influenza A/H7N9. Time scale phylogenetic trees of PB2, PB1, PA, HA, NP, NA, M1 and NS1 genes of influenza H7N9 strain.

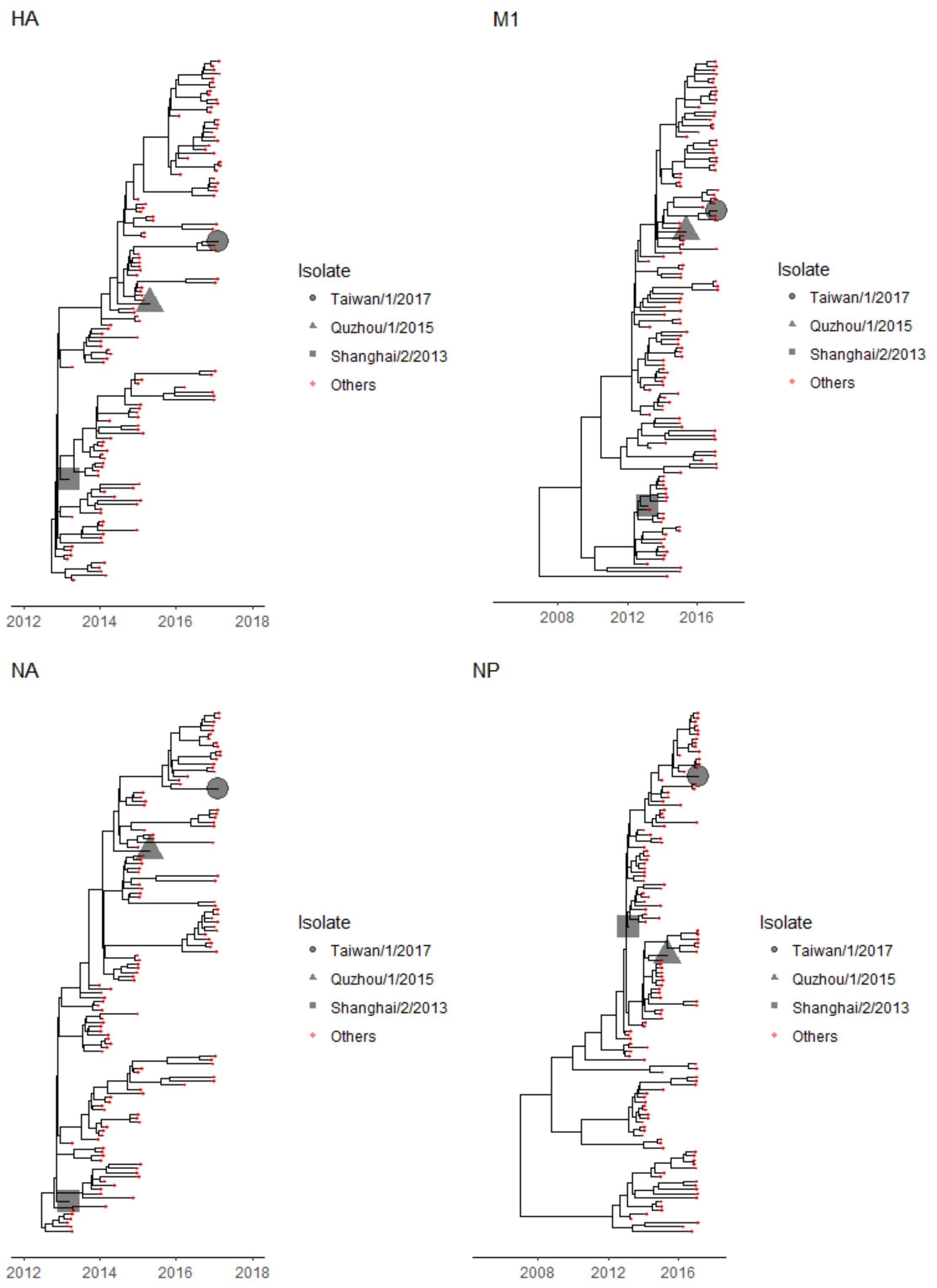

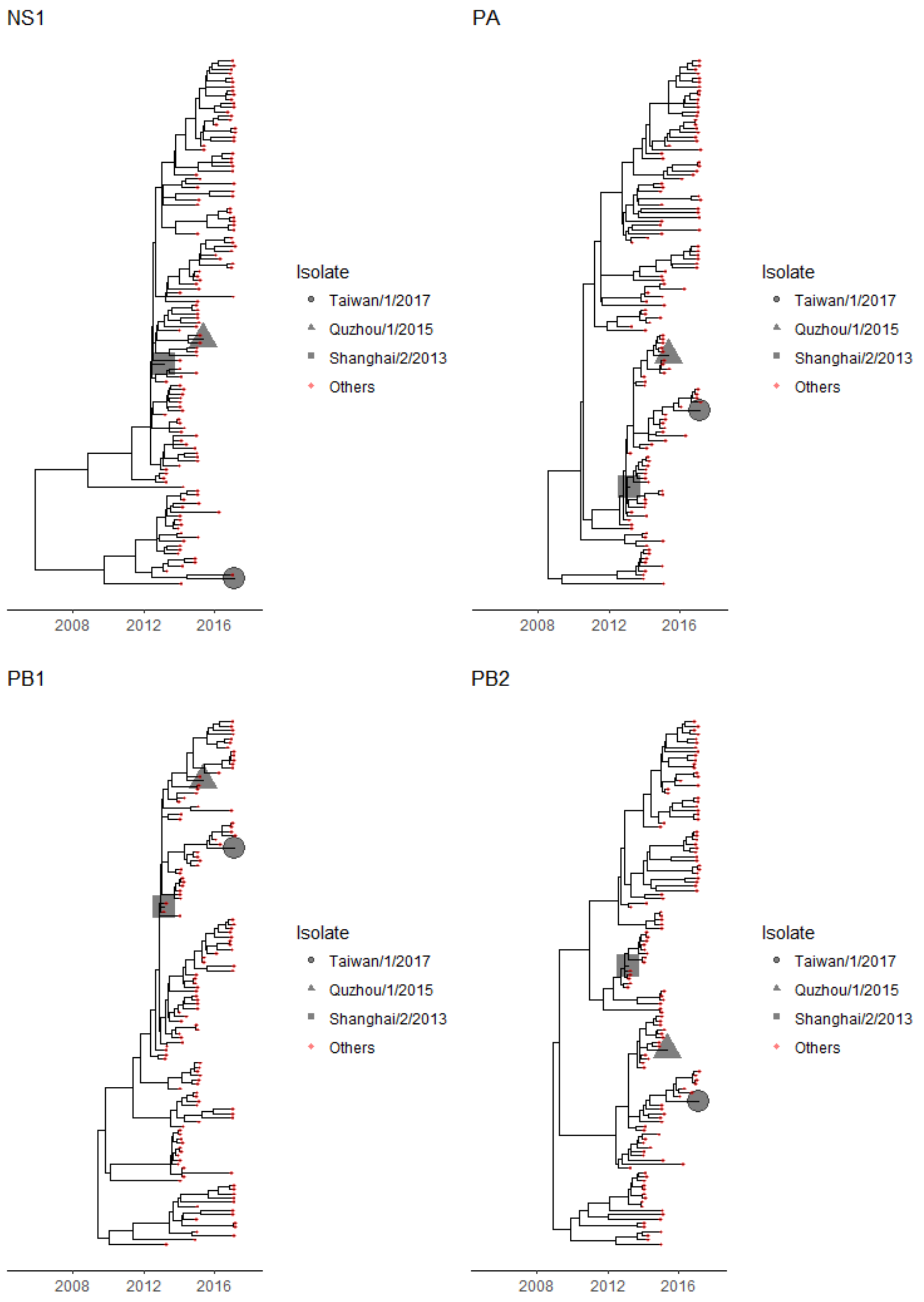
NS1
PA
PB1
PB2
Isolate
Taiwan/1/2017
Quzhou/1/2015
Shanghai/2/2013
Others
2008
2012
2016
