# Supplementary material for: Computational analysis of the receptor binding specificity of novel influenza A/H7N9 viruses
Source: BMC Genomics. 2018 May 9;19(Suppl 2):88. doi: 10.1186/s12864-018-4461-z (PMC5954268; doi:10.1186/s12864-018-4461-z)

Additional file 2: Superimpose the best and worst docked HA-ligand complexes.

Visualize the structure of docked SH13-LSTa, SH13-LSTc, TW17-LSTa and TW17-LSTc complexes with the highest and the lowest binding affinities.

1. The optimal complex SH13-LSTa with score had SIA towards the 220-loop (colored pink), while the worst case had SIA towards the 130-loop (colored blue). The binding affinity of the best and worst docked complex was -6.3 kcal/mol and -5.3 kcal/mol respectively.

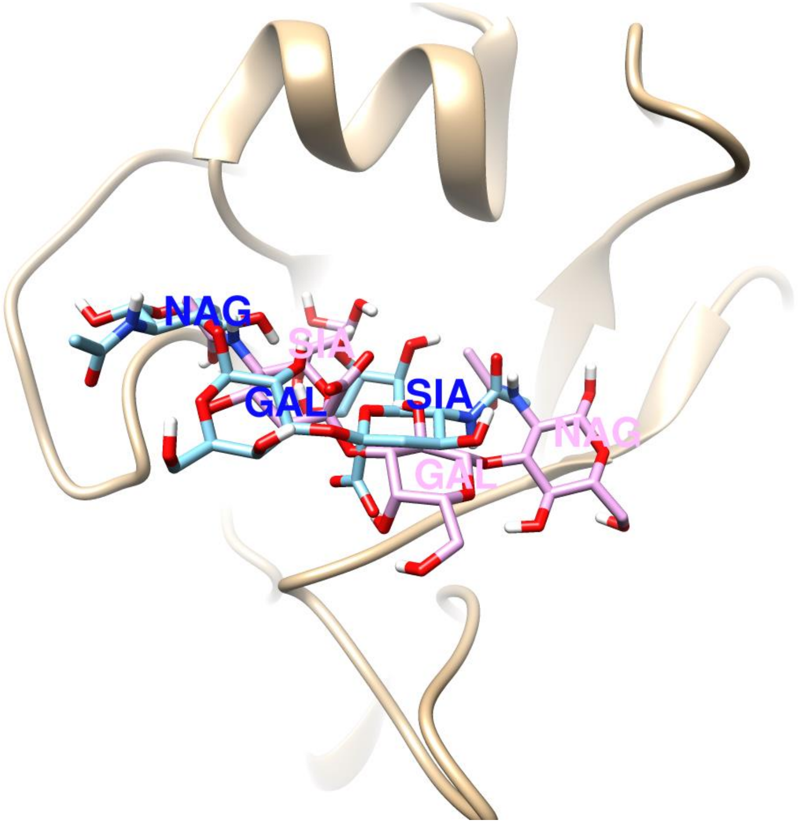

2. The optimal complex TW17-LSTa with score had SIA towards the 220-loop (colored pink), while the worst case had SIA towards the 130-loop (colored blue). The binding affinity of the best and worst docked complex was -6.3 kcal/mol and -4.8 kcal/mol respectively.

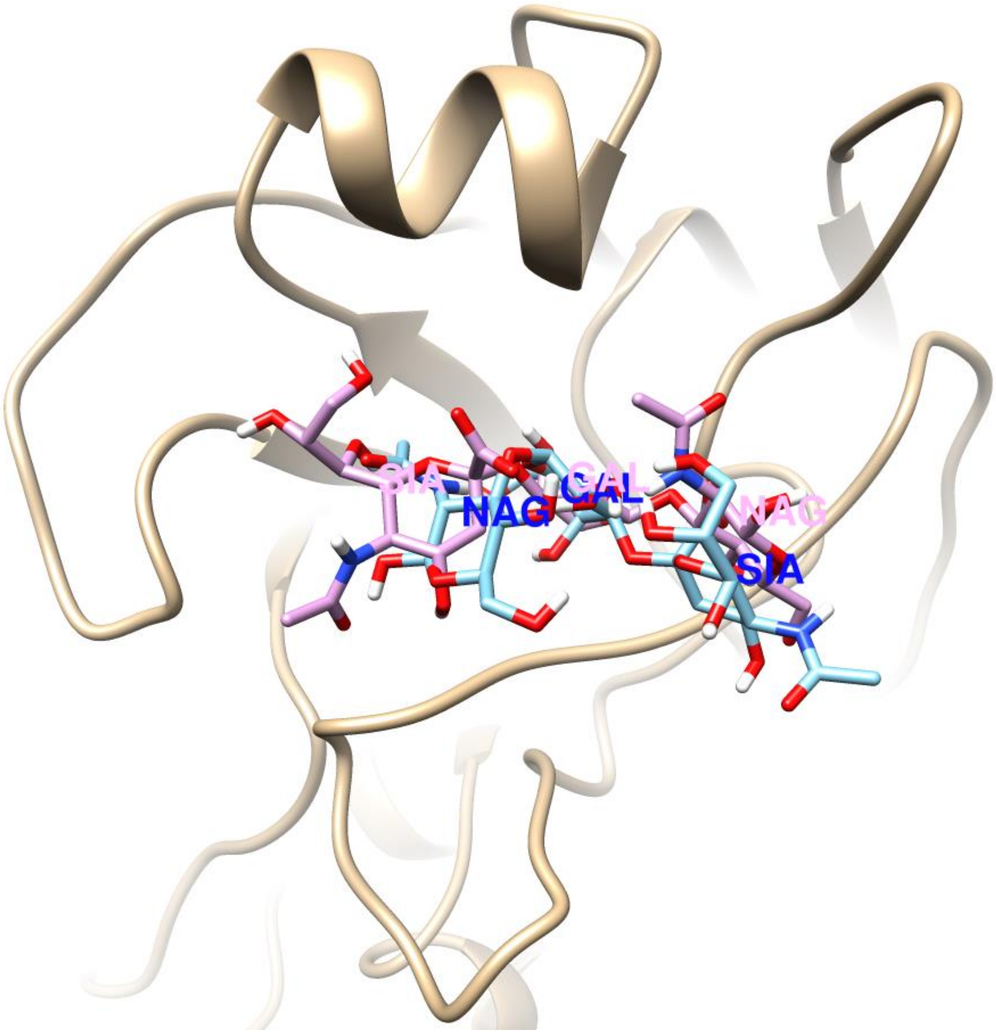

3. The optimal complex SH13-LSTc with score had SIA towards the 130-loop (colored pink), while the worst case had SIA towards the 220-loop (colored blue). The binding affinity of the best and worst docked complex was -6.3 kcal/mol and -5.0 kcal/mol respectively.

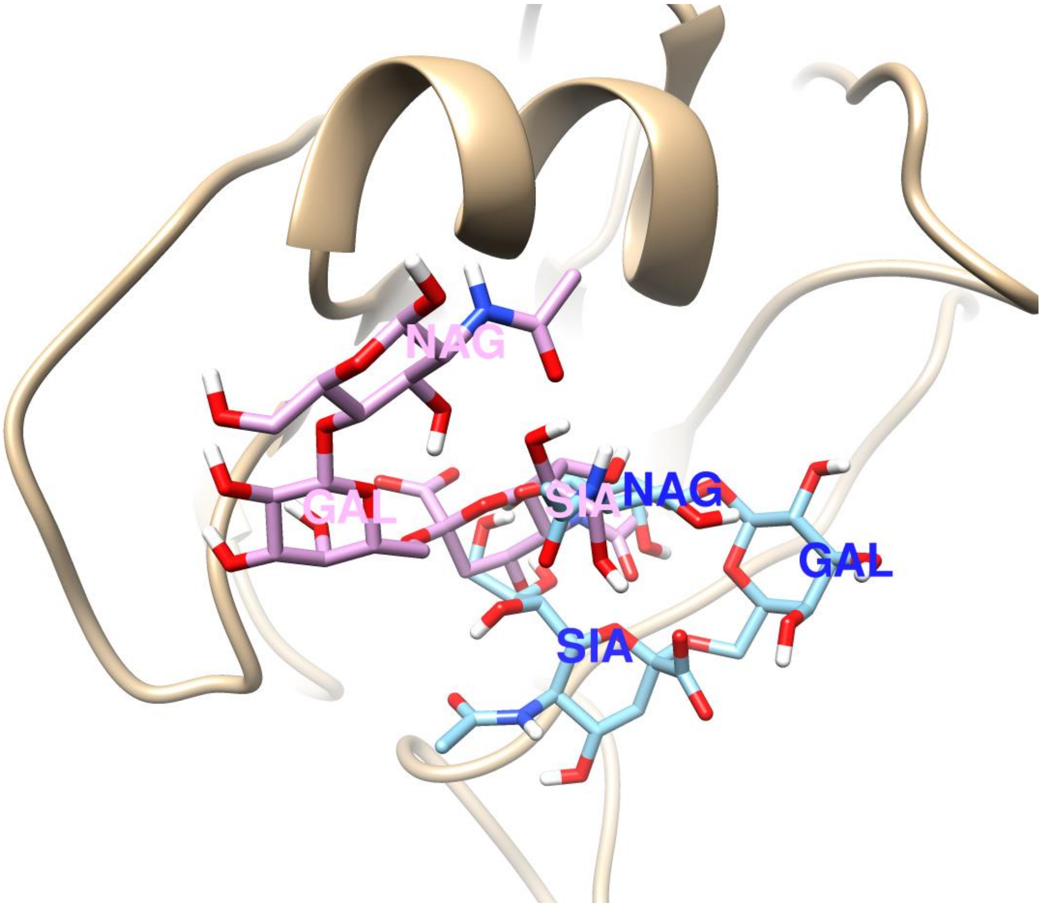

4. The optimal complex TW17-LSTc with score had SIA towards the 130-loop (colored pink), while the worst case had SIA towards the 220-loop (colored blue). The binding affinity of the best and worst docked complex was -6.8 kcal/mol and -5.6 kcal/mol respectively.

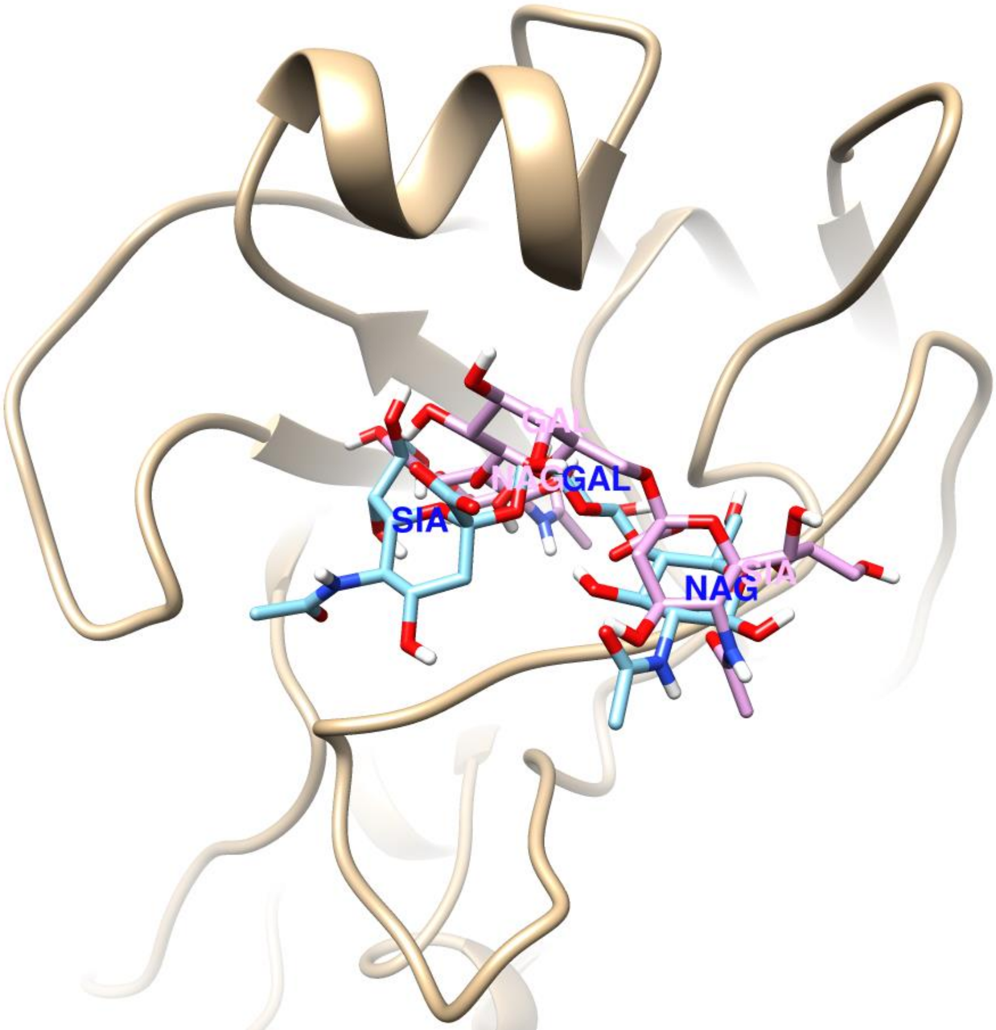

Supplement: Supplementary file 2 — Superimpose the best and worst docked HA-ligand complexes. Visualize the structure of docked SH13-LSTa, SH13-LSTc, TW17-LSTa and TW17-LSTc complexes with the highest and the lowest binding affinities. (PDF 466 kb) [file 12864_2018_4461_MOESM2_ESM.pdf]
