# Supplementary material for: Computational analysis of the receptor binding specificity of novel influenza A/H7N9 viruses
Source: BMC Genomics. 2018 May 9;19(Suppl 2):88. doi: 10.1186/s12864-018-4461-z (PMC5954268; doi:10.1186/s12864-018-4461-z)

Additional file 3. VdW energy, electrostatic energy and total interaction energy of SH13-LSTa, SH13-LSTc, TW17-LSTa and TW17-LSTc during the whole simulation (50 ns).

The total energy of TW17-LSTa fluctuated obviously during around 25 ns. Each trajectory fluctuated within a small range after 30ns. Therefore, we chose the last 20 ns frames for further analysis.

1. SH13-LSTa

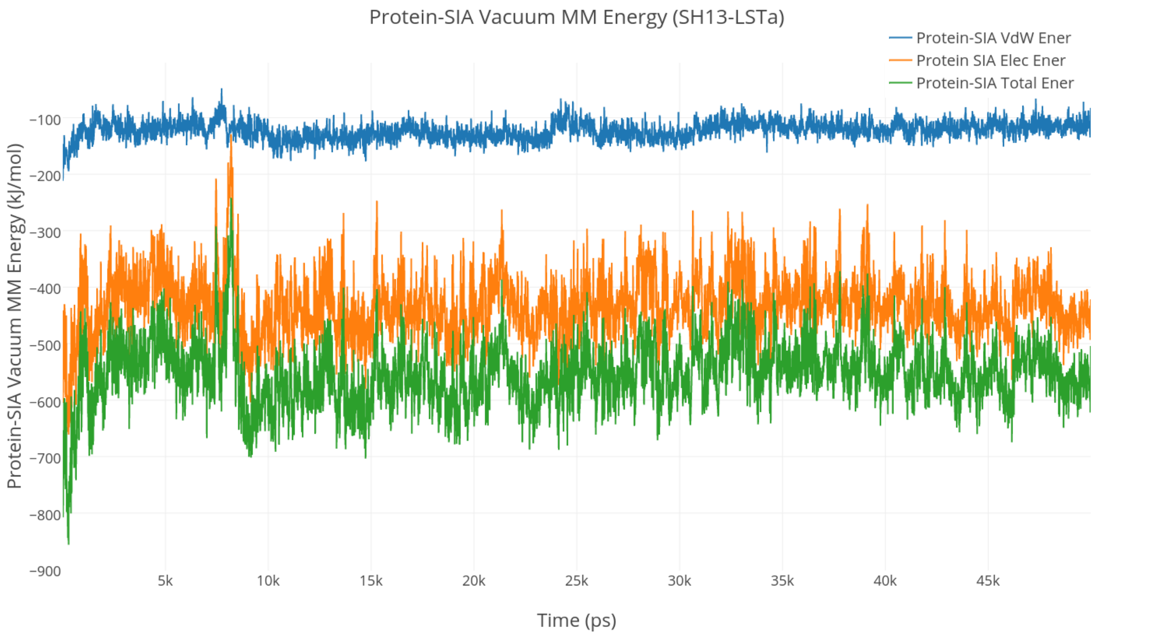

2. SH13-LSTc

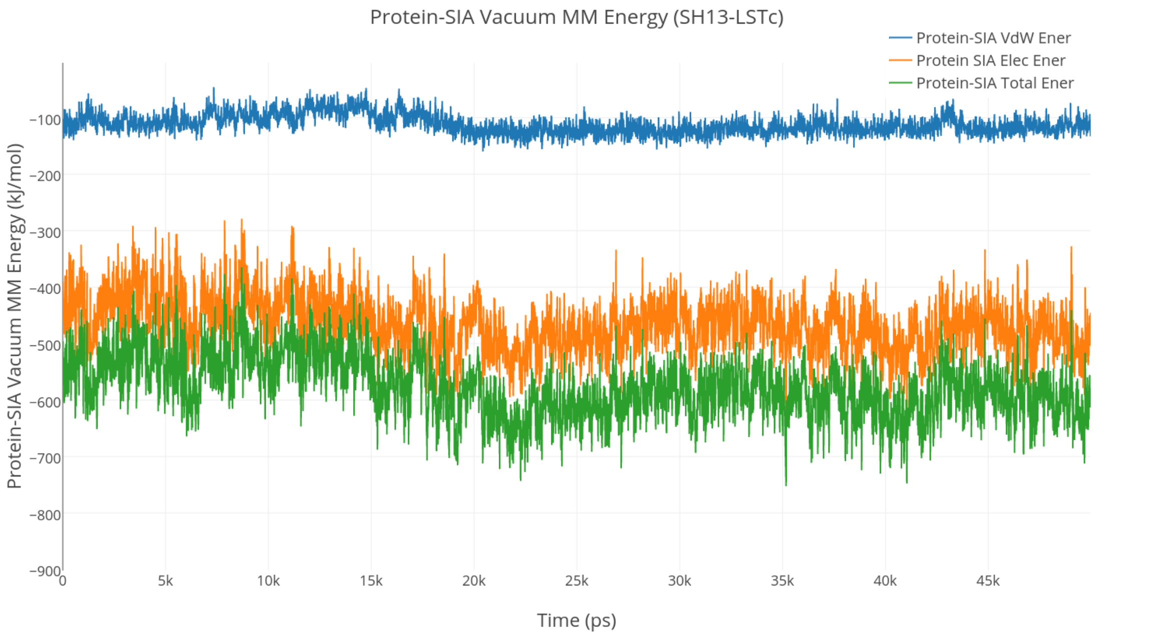

3. TW17-LSTa

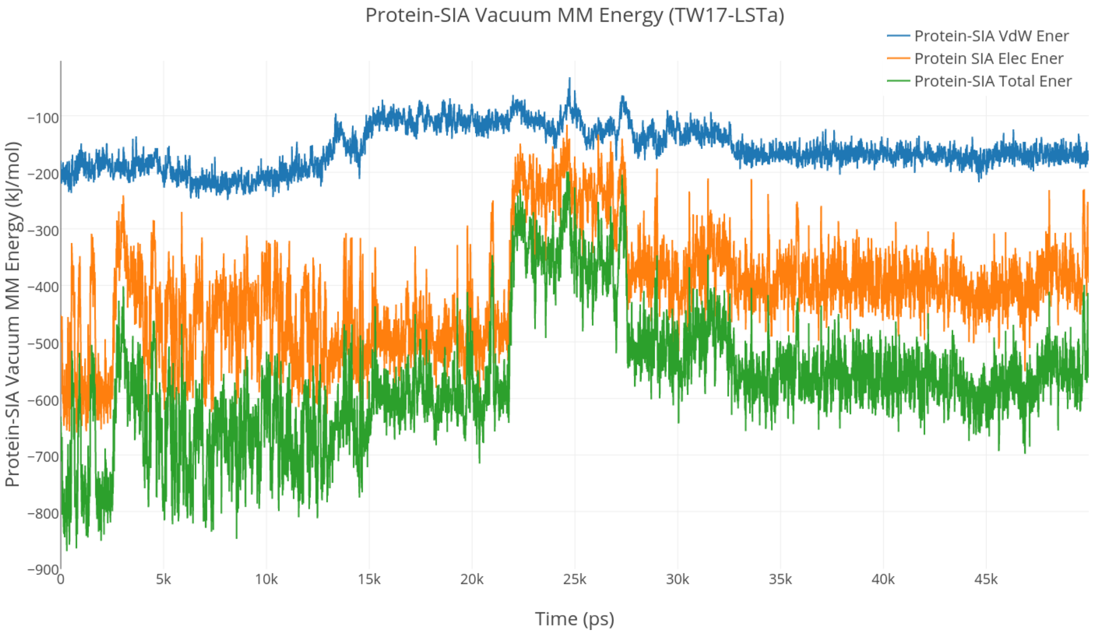

4. TW17-LSTc

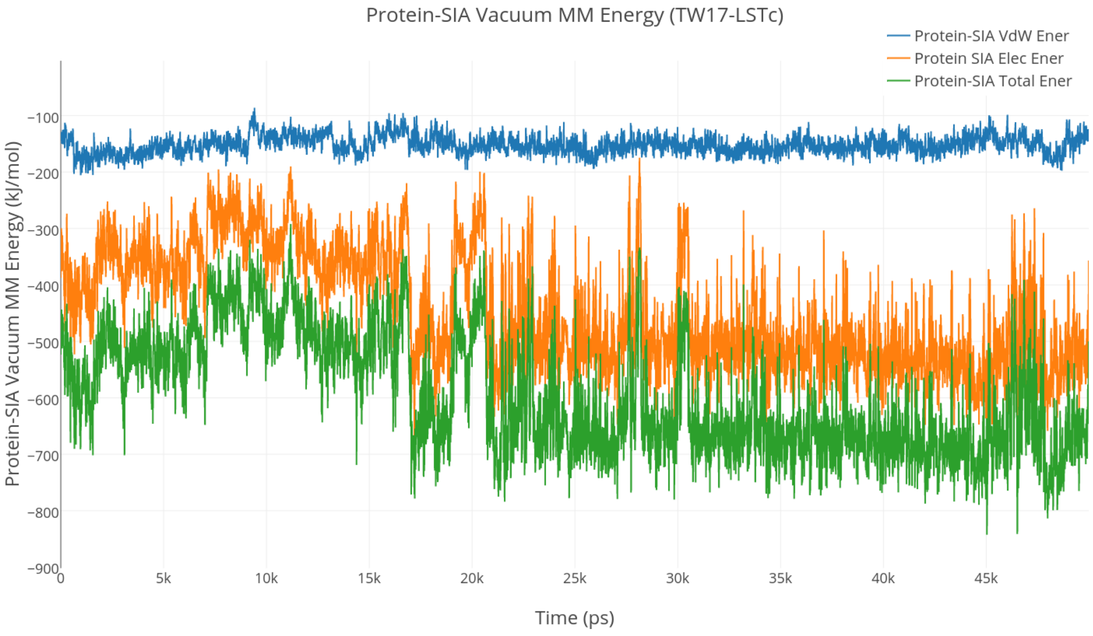

Supplement: Supplementary file 3 — Energy decomposition of HA-ligand complexes. VdW energy, electrostatic energy and total interaction energy of SH13-LSTa, SH13-LSTc, TW17-LSTa and TW17-LSTc. (PDF 702 kb) [file 12864_2018_4461_MOESM3_ESM.pdf]
