# Supplementary material for: Computational analysis of the receptor binding specificity of novel influenza A/H7N9 viruses
Source: BMC Genomics. 2018 May 9;19(Suppl 2):88. doi: 10.1186/s12864-018-4461-z (PMC5954268; doi:10.1186/s12864-018-4461-z)
Supplement: Supplementary file 4 — Two rounds of 50 ns molecular dynamics simulation for SH13-LSTa, SH13-LSTc, TW17-LSTa, TW17-LSTc. Average total binding energy (kJ/mol) of the HA-LSTa/LSTc complexes. (PDF 424 kb) [file 12864_2018_4461_MOESM4_ESM.pdf]

Additional file 4. Two rounds of 50 ns molecular dynamics simulation for SH13-LSTa, SH13-LSTc, TW17-LSTa, TW17-LSTc

we conducted two rounds 50 ns molecular dynamics simulation. The total binding energy for each system has been shown in the following figures.

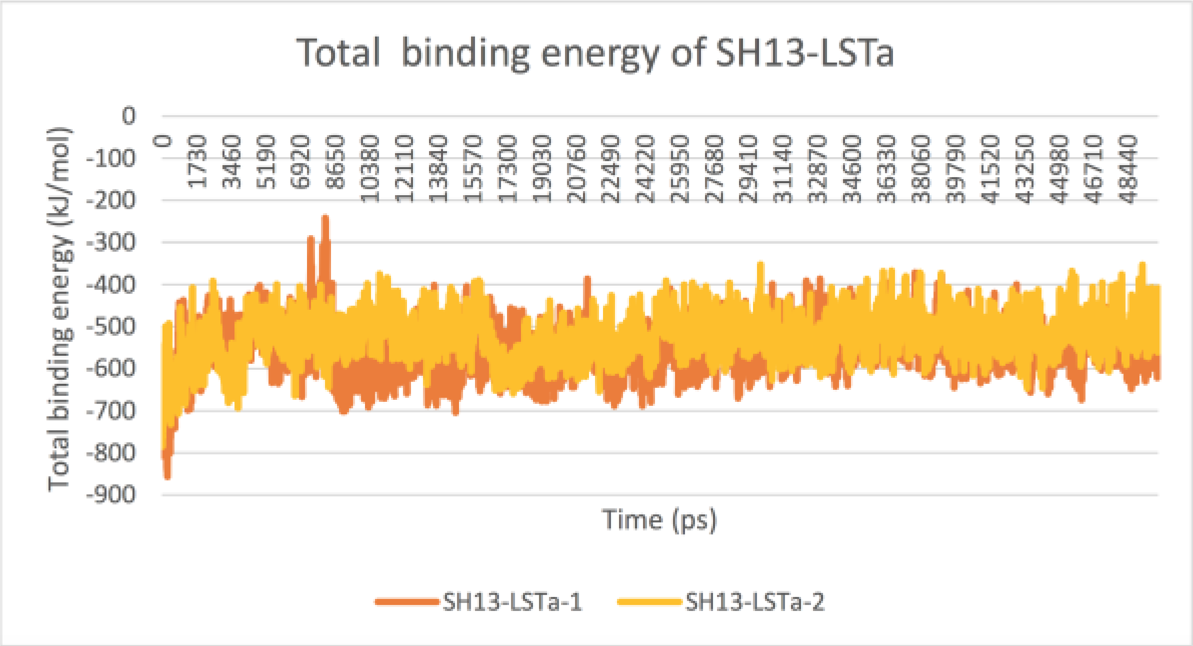

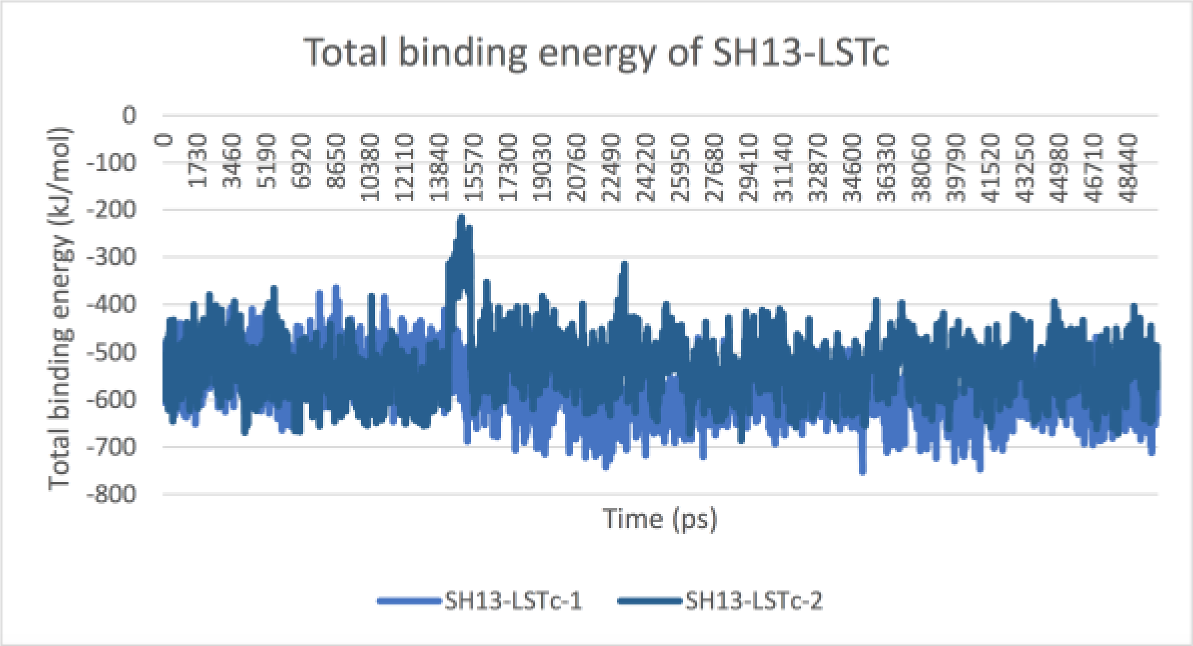

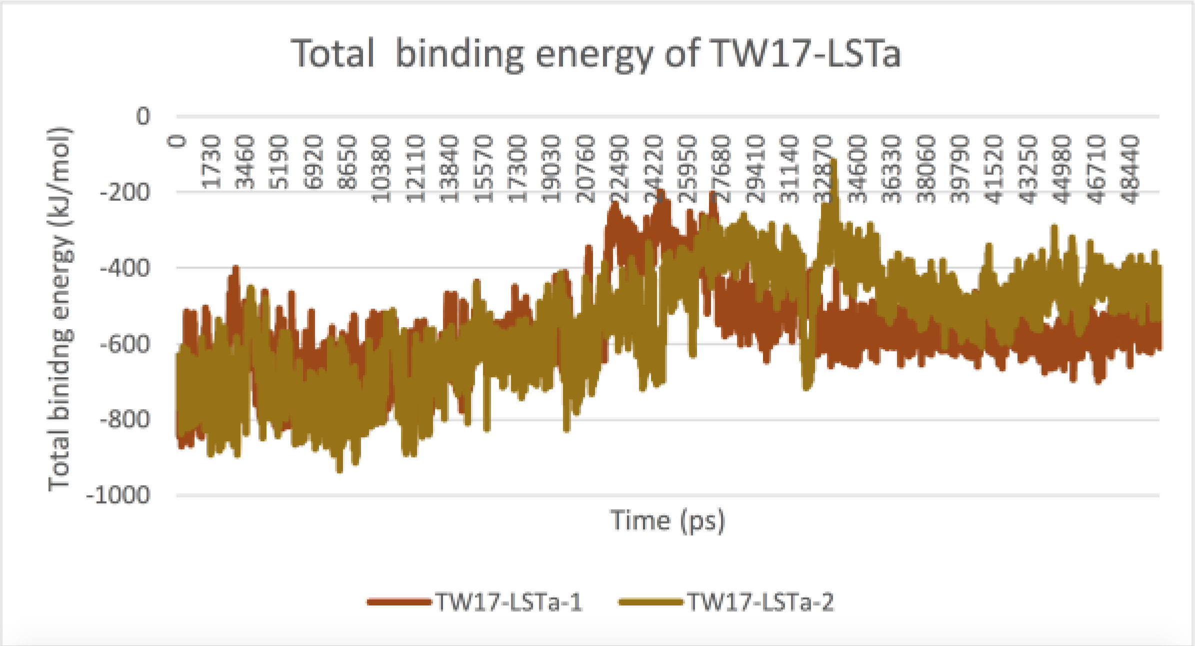

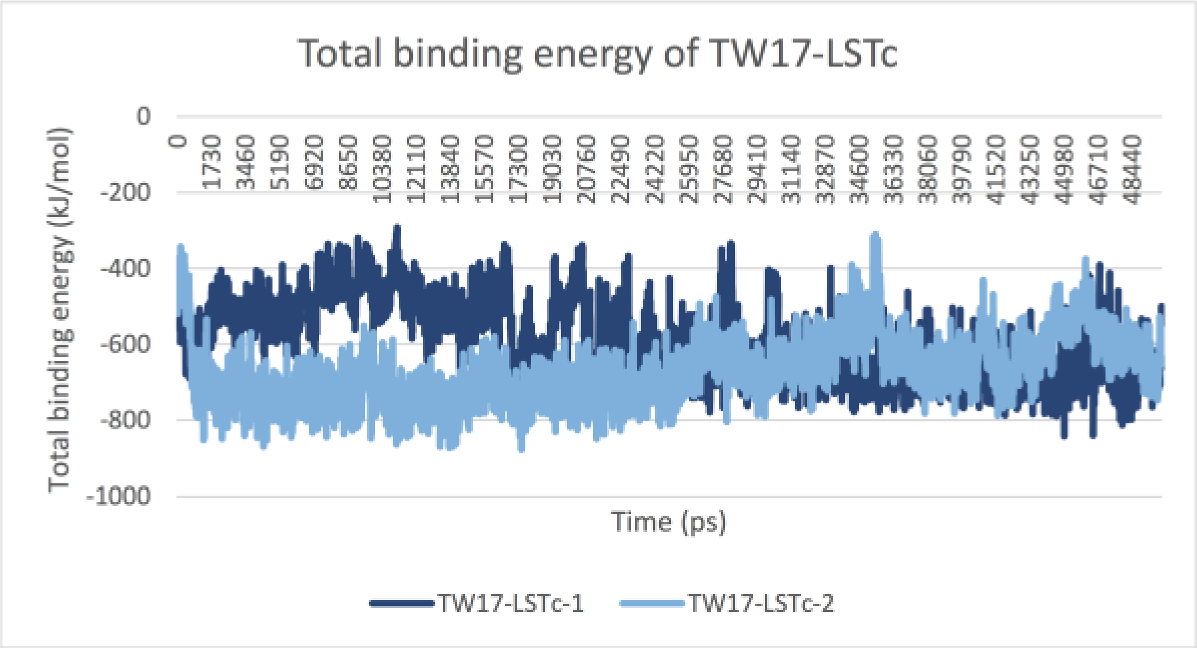

The averages of total binding energy for the HA-LSTa/LSTc complexes are listed in Table 1. The results are consistent with the main text. The mutant TW17 HA obtains the largest binding energy, and enhances the binding with two types receptors, especially LSTc. Both SH13 and TW17 strains have binding preference to LSTc. Additional file 6 shows the simulation results of each system and the average total binding energy.

Table 1. Average total binding energy (kJ/mol) of the HA-LSTa/LSTc complexes.

	**LSTa**	**LSTc**	**[1]ΔE_1**
**SH13**	-539.168	-554.499	+15.331
**TW17**	-557.506	-633.218	+75.712
**[2]ΔE_2**	+18.338	+78.719	

1 Binding preference of HA protein: $\Delta E_1 = \Delta E_{HA,\ LSTa} - \Delta E_{HA,\ LSTc}$

2 Difference of HAs binding to receptors: $\Delta E_2 = \Delta E_{SH13,\ receptor} - \Delta E_{TW17,\ receptor}$
