# Supplementary material for: Computational analysis of the receptor binding specificity of novel influenza A/H7N9 viruses
Source: BMC Genomics. 2018 May 9;19(Suppl 2):88. doi: 10.1186/s12864-018-4461-z (PMC5954268; doi:10.1186/s12864-018-4461-z)

**Additional file 5. Residue contributions during the whole simulation process.**

**1. Energy contribution of the residues involved in interaction in the complex SH13-LSTa**

For most residues, the residue-ligand interaction energy fluctuated within a small range, except S145 and S152.

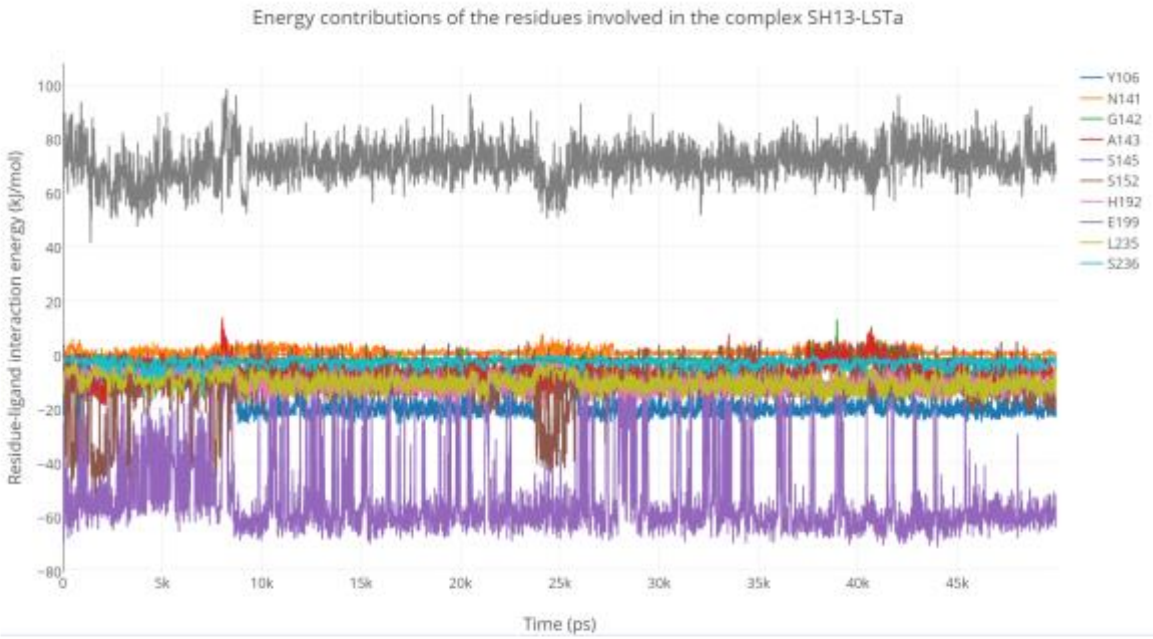


**2. Energy contribution of the residues involved in interaction in the complex SH13-LSTc.**

The residue-ligand interaction energy fluctuated within 10 kJ/mol after 20 ns.

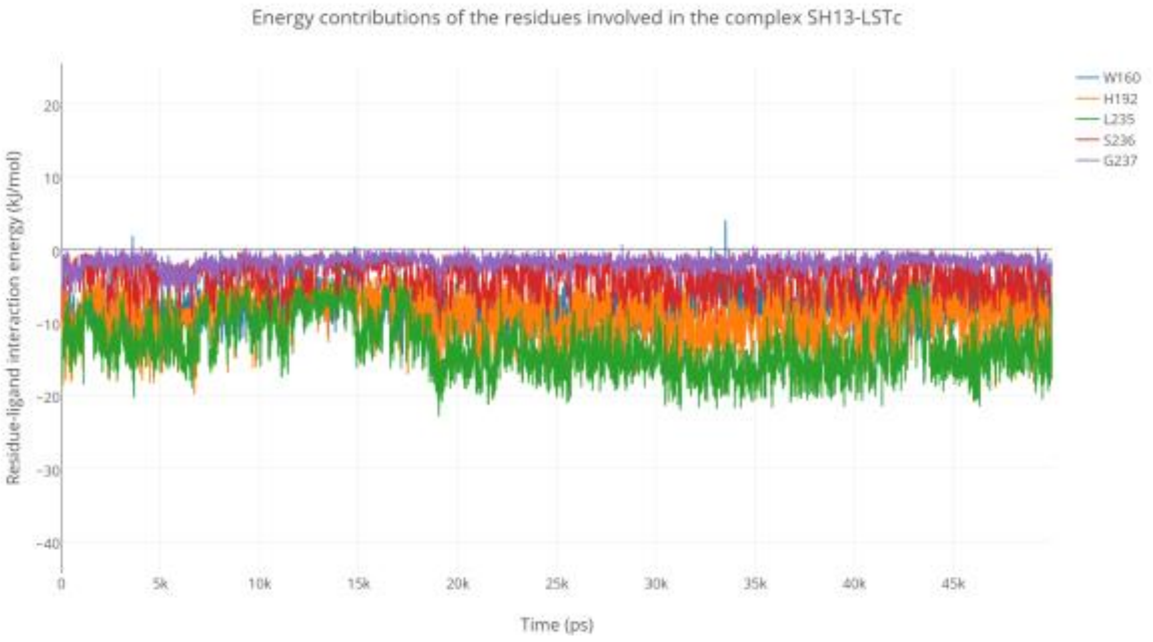

**3. Energy contribution of the residues involved in interaction in the complex TW17-LSTa.**

For most residues, the residue-ligand interaction energy fluctuated within a small range for the whole simulation process, except R139, which converged after 28 ns.

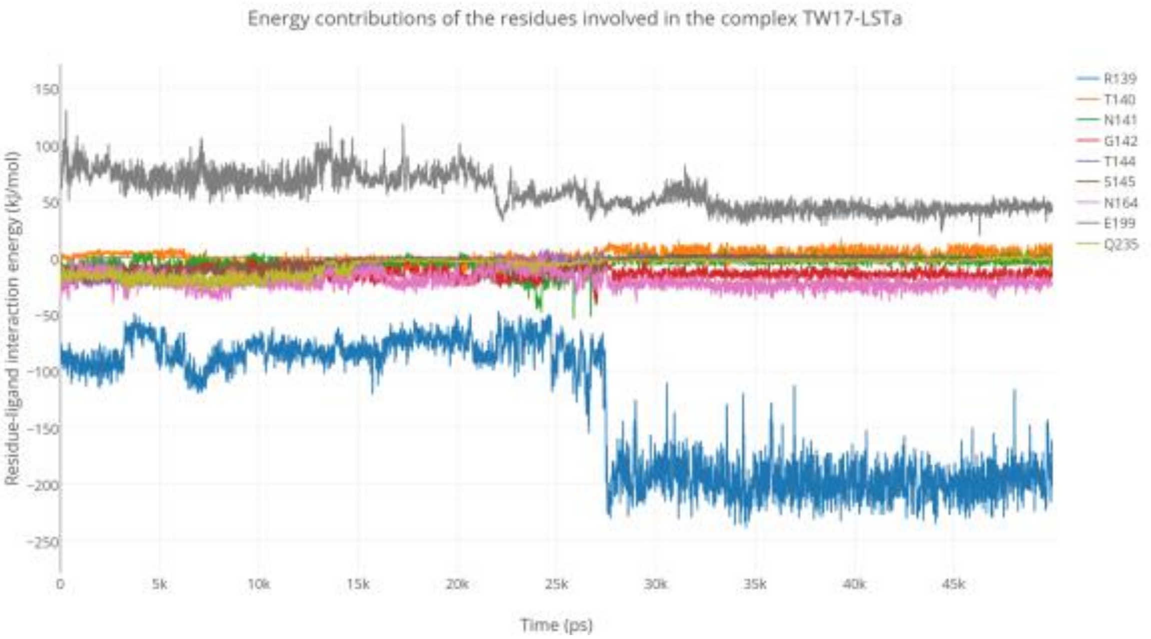


**4. Energy contribution of the residues involved in interaction in the complex TW17-LSTc.**

The residue-ligand interaction energy fluctuated within a small range. The fluctuation for R139 is slightly larger than the others.

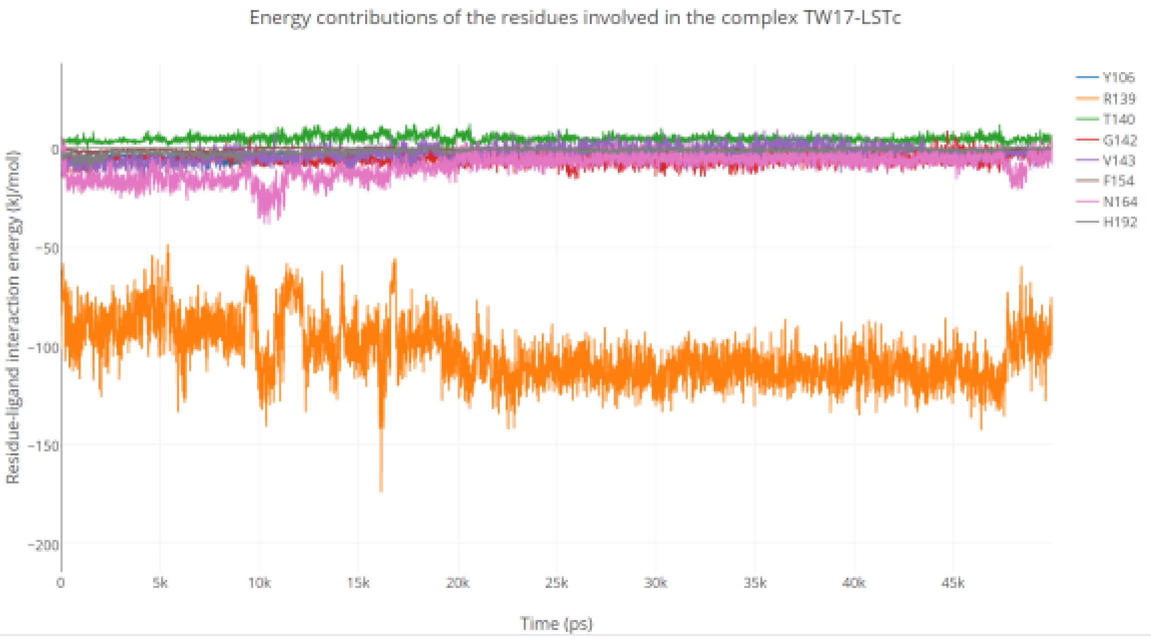

Supplement: Supplementary file 5 — Residues contribution during the whole simulation process. The convergence analysis of energy contribution for residues involved in HA-ligand interaction. (PDF 153 kb) [file 12864_2018_4461_MOESM5_ESM.pdf]
