# Supplementary material for: Computational analysis of the receptor binding specificity of novel influenza A/H7N9 viruses
Source: BMC Genomics. 2018 May 9;19(Suppl 2):88. doi: 10.1186/s12864-018-4461-z (PMC5954268; doi:10.1186/s12864-018-4461-z)

Additional file 6. Average energy contribution of residues that involved in receptor-ligand interactions in the optimally docked complexes

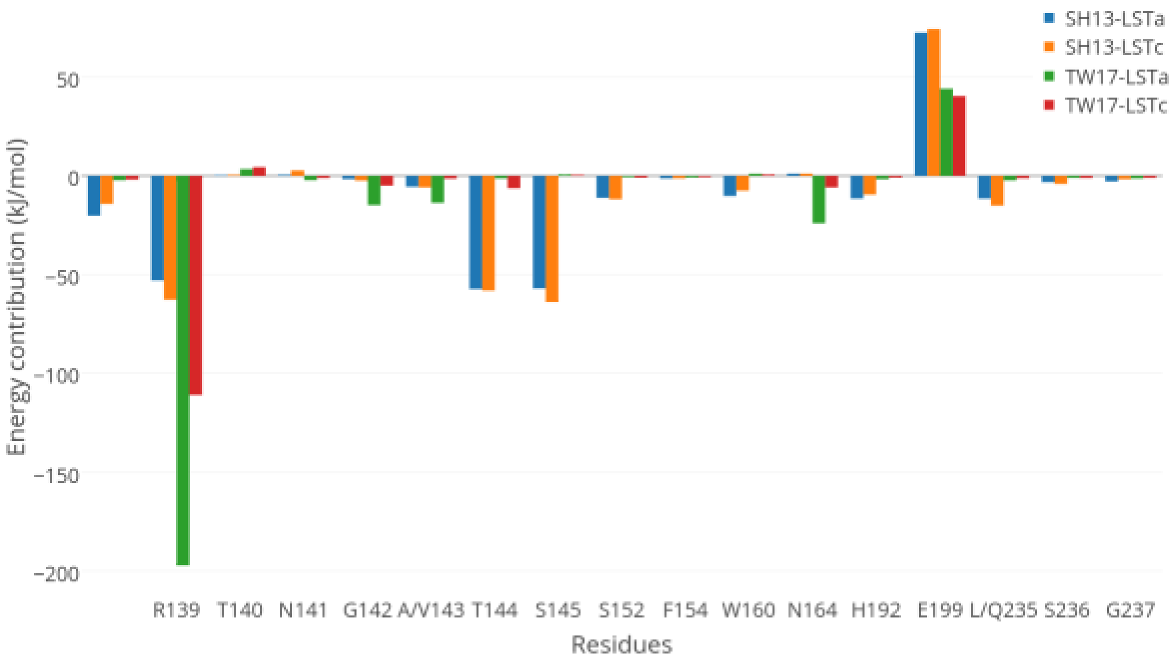

Supplement: Supplementary file 6 — Average energy contribution of residues that involved in receptor-ligand interactions in the optimally docked complexes. (PDF 24 kb) [file 12864_2018_4461_MOESM6_ESM.pdf]
